# Supplementary material for: Translating GWAS-identified loci for cardiac rhythm and rate using an in vivo image- and CRISPR/Cas9-based approach
Source: Sci Rep. 2020 Jul 16;10:11831. doi: 10.1038/s41598-020-68567-1 (PMC7367351; doi:10.1038/s41598-020-68567-1)
Supplement: Supplementary file 1 — Supplementary Information 1. [file 41598_2020_68567_MOESM1_ESM.pdf]

Translating GWAS-identified loci for cardiac rhythm and rate using an *in vivo* image- and CRISPR/Cas9-based approach

Benedikt von der Heyde[1,2#], Anastasia Emmanouilidou[1,2#], Eugenia Mazzaferro[1,2], Silvia Vicenzi[1,2], Ida Höijer[1,2], Tiffany Klingström[2,3], Sitaf Jumaa[1,2], Olga Dethlefsen[4,5], Harold Snieder[6], Eco de Geus[7], Adam Ameur[1,2,8], Erik Ingelsson[2,9,10,11], Amin Allalou[2,12], Hannah L. Brooke[13], Marcel den Hoed[1,2*]

# denotes equal contribution

SUPPLEMENTARY INFORMATION

<u>SUPPLEMENTARY FIGURES</u>

Supplementary Figure 1 – Experimental pipeline (page 3)

Supplementary Figure 2 – Flow chart (page 4)

Supplementary Figure 3 – Distribution of heart rate variability and heart rate in the CRISPR/Cas9 experiment (page 5-10)

Supplementary Figure 4 – Distribution of heart rate variability and heart rate in the CRISPR/Cas9 experiment (page 11-21)

Supplementary Figure 5 – Alignment of CRISPR/Cas9-induced mutations in the nine candidate genes (page 22-23)

Supplementary Figure 6 – Distribution of mutated alleles across the nine candidate genes (page 24)

Supplementary Figure 7 – Effect of mutations in the nine candidate genes on body size (page 25)

Supplementary Figure 8 – Off-target mutagenic activity for *hnc4*, *hcn4l*, *neo1a* and *neo1b* gRNAs examined using Nanopore off-target sequencing (page 26)

Supplementary Figure 9 – Distribution heart rate variability and heart rate in ivabradine experiment (page 27-28)

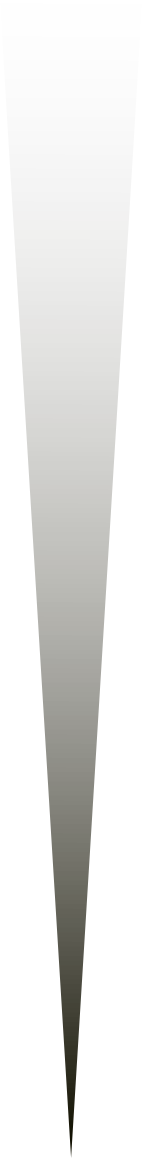

Eight loci associated with HRV in humans[1].
18 genes prioritized for functional follow-up[1].

Six human genes selected for follow up,
represented by nine orthologues in zebrafish.

CRISPR-Cas9 gRNA design,
synthesis and efficiency testing.

Raising of CRISPR-Cas9 founders (F_0).
Crossing of F_0 generation to yield F1 embryos.

Acquisition of 30s recordings of the beating atrium
in 381 live, intact F_1 embryos at 2dpf and at 5dpf.

Automated quantification of cardiac traits in 30s
recordings using custom-written MatLab code.

Paired-end sequencing of CRISPR-targeted
sites using a MiSeq (2x250bp).

Splitting, merging, QC-ing, aligning
in sequencing data.

Transcript- and allele-specific variant calling using
a custom-written algorithm.

Variant annotation using Ensembl's variant effect predictor.

Genetic association analysis using hierarchical linear
models and multiple logistic regression analyses.

Supplementary Figure 1: Experimental pipeline describing the workflow from GWAS in humans to stastistical analysis in zebrafish embryos. dpf: days post-fertilization.

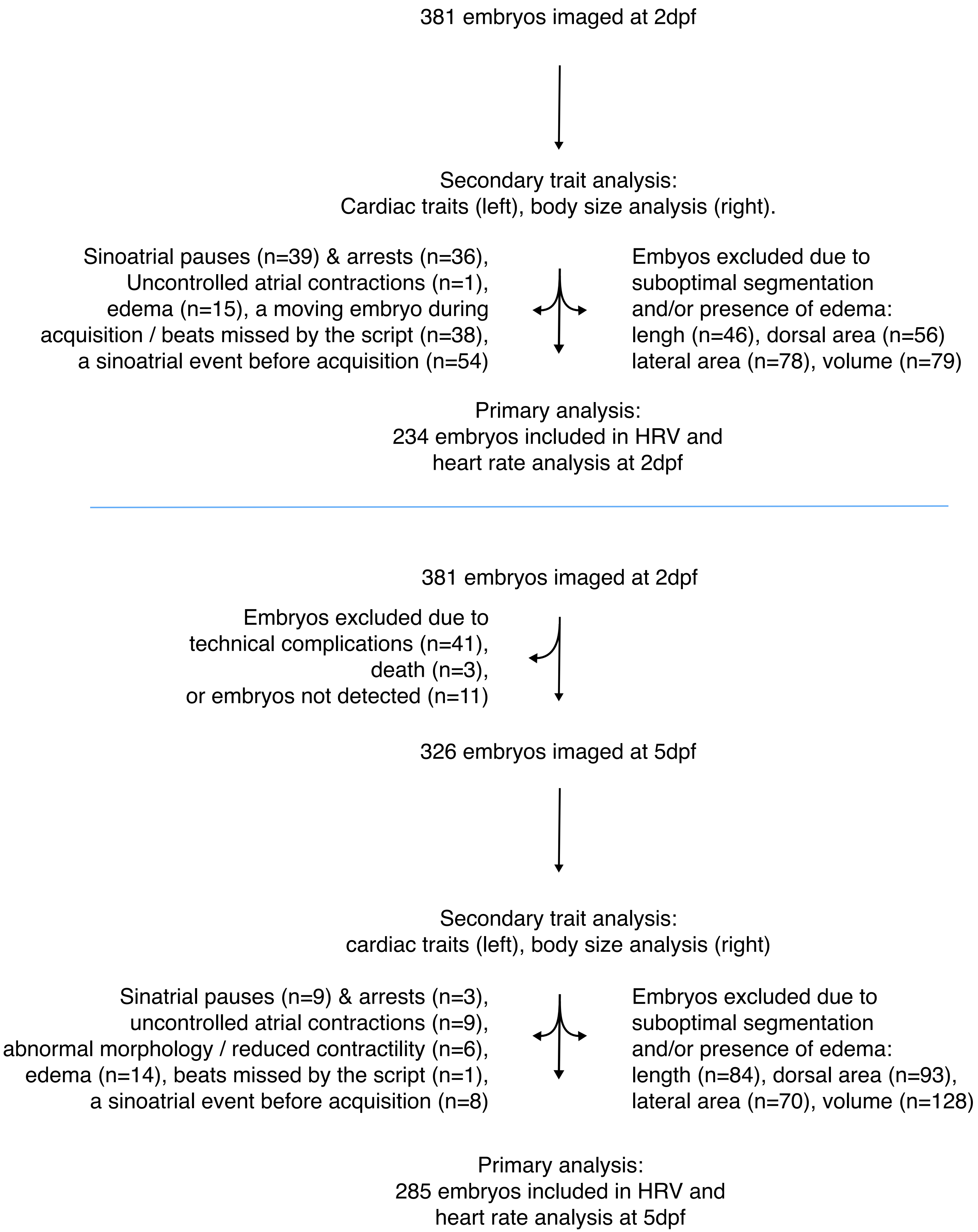

Supplementary Figure 2: Flow chart showing the number of embryos included in the analysis and reasons for exclusion. Embryos can be excluded for more than one reason.

Overall distribution

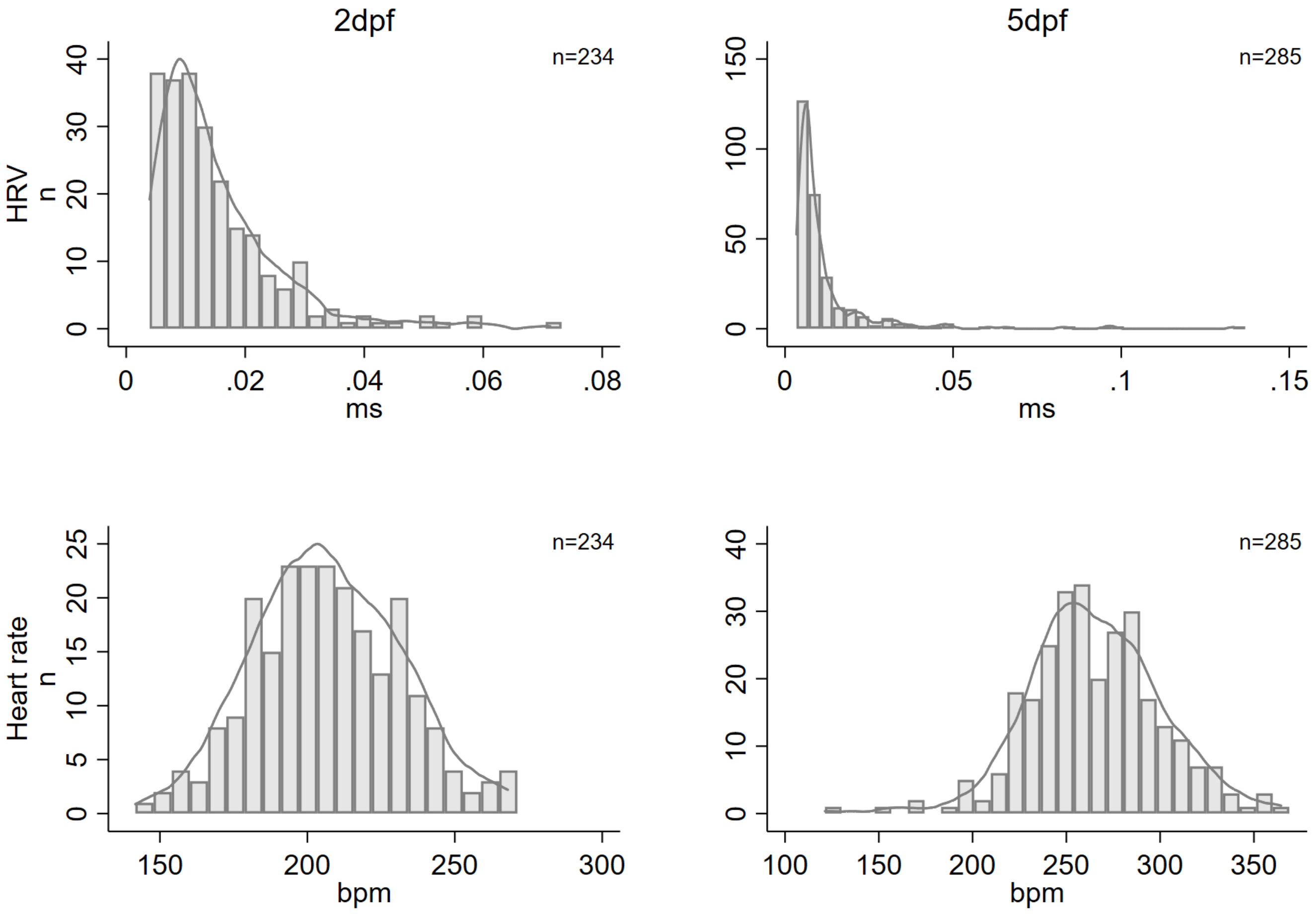

gngt1

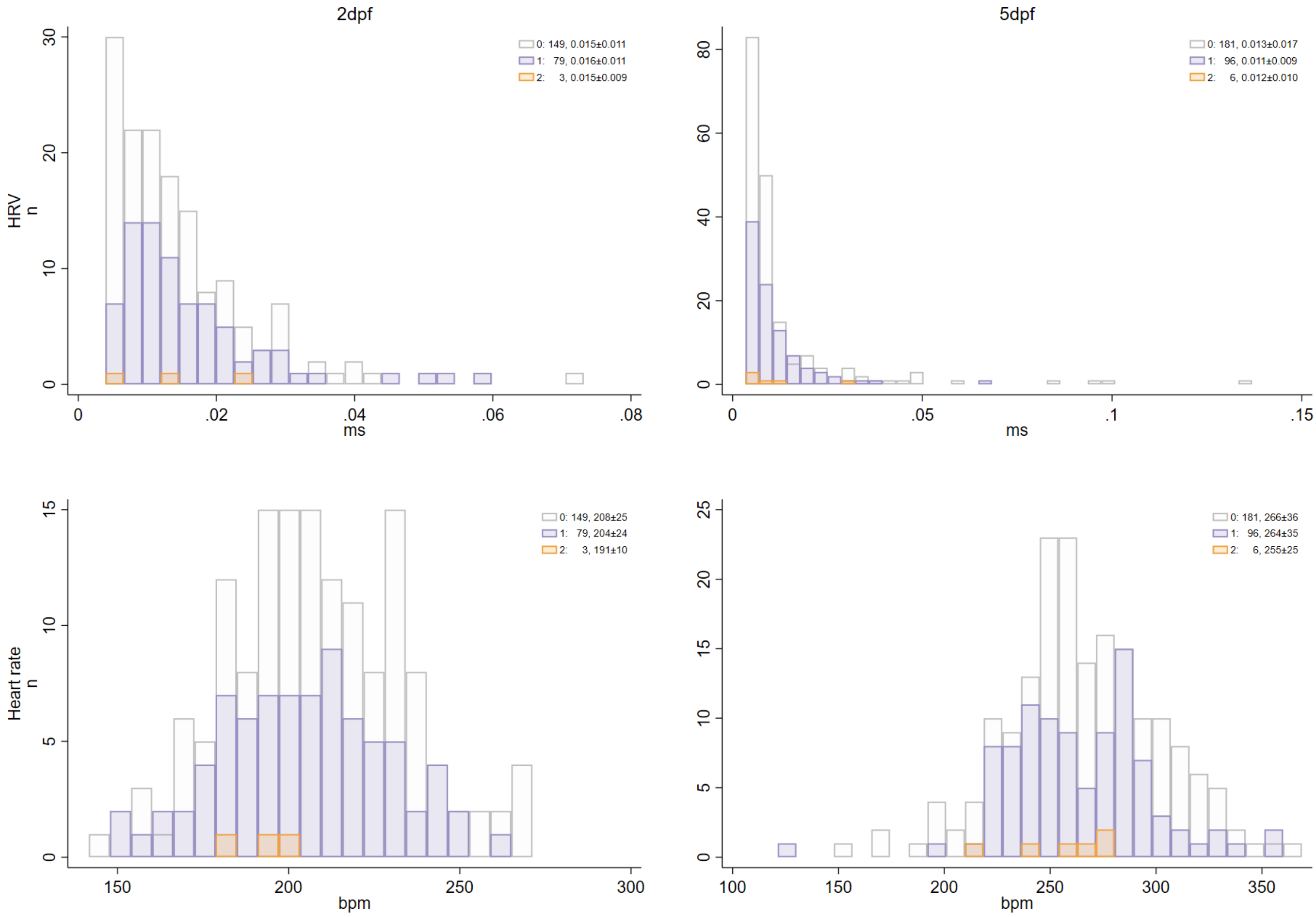

syt10

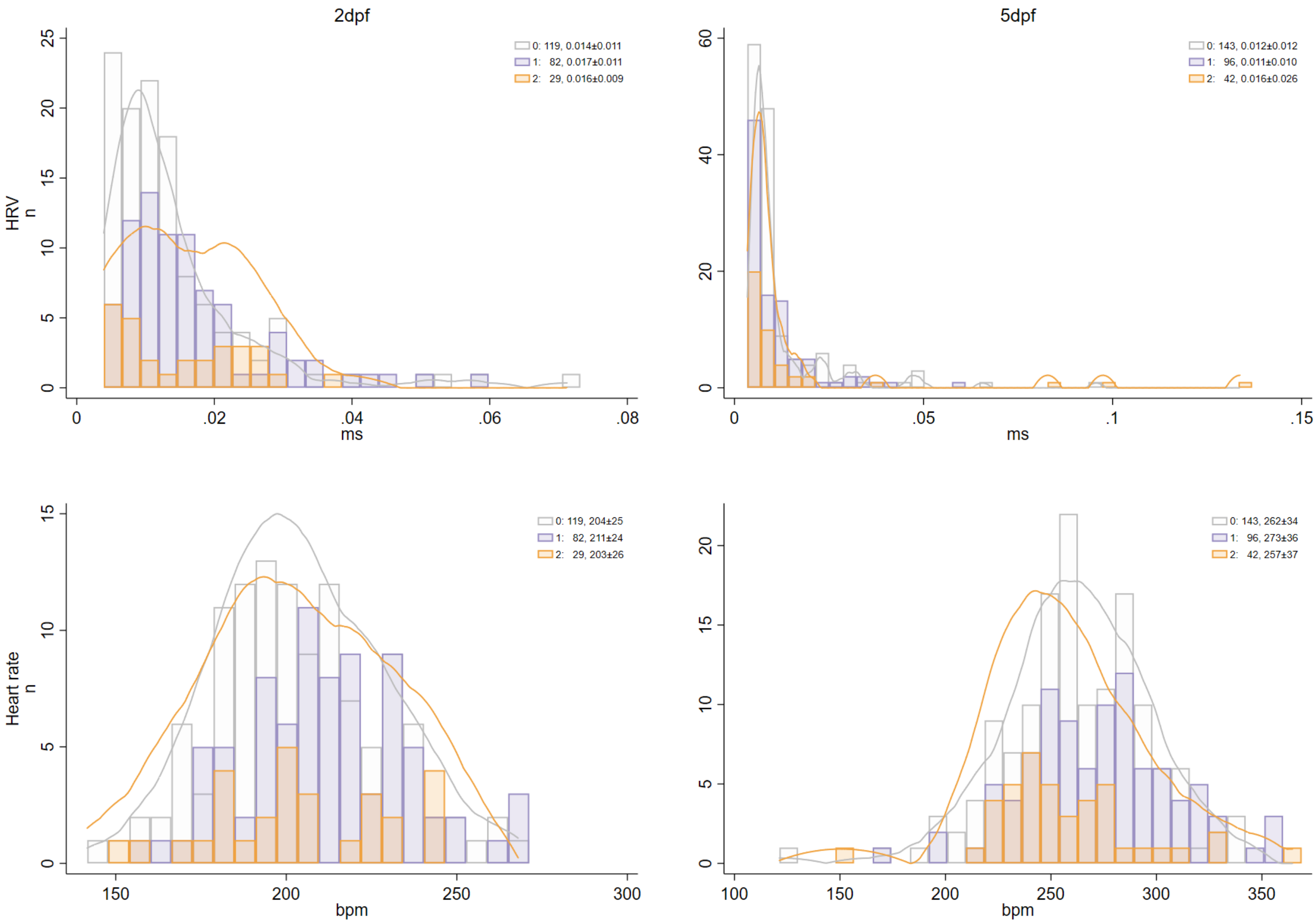
2dpf
5dpf
HRV
n
ms
Heart rate
bpm
0: 119, 0.014±0.011
1: 82, 0.017±0.011
2: 29, 0.016±0.009
0: 143, 0.012±0.012
1: 96, 0.011±0.010
2: 42, 0.016±0.026
0: 119, 204±25
1: 82, 211±24
2: 29, 203±26
0: 143, 262±34
1: 96, 273±36
2: 42, 257±37

rgs6

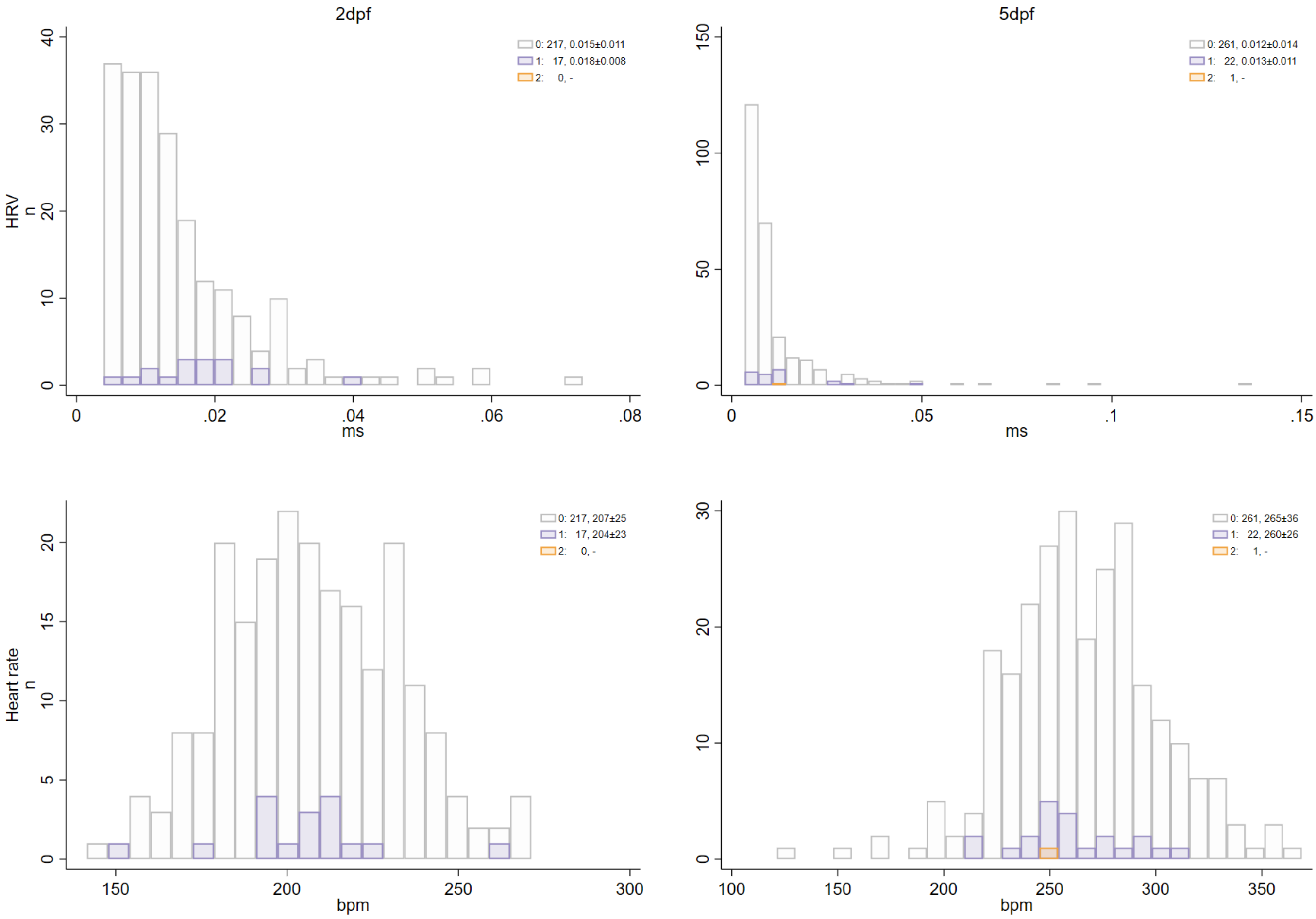
2dpf
5dpf
HRV
n
ms
Heart rate
bpm
0: 217, 0.015±0.011
1: 17, 0.018±0.008
2: 0, -
0: 261, 0.012±0.014
1: 22, 0.013±0.011
2: 1, -
0: 217, 207±25
1: 17, 204±23
2: 0, -
0: 261, 265±36
1: 22, 260±26
2: 1, -

hcn4

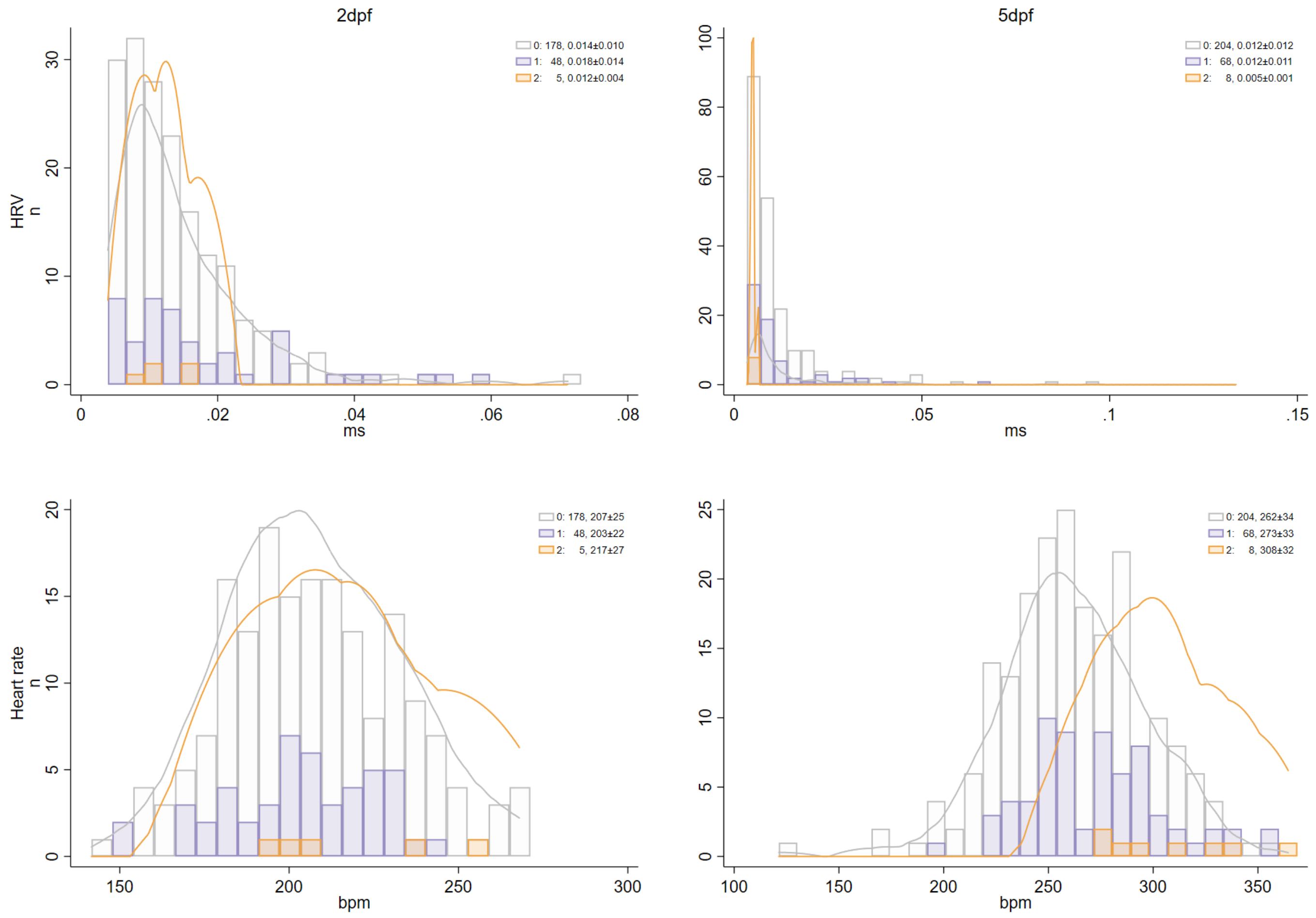
2dpf
0: 178, 0.014±0.010
1: 48, 0.018±0.014
2: 5, 0.012±0.004
HRV
n
ms
5dpf
0: 204, 0.012±0.012
1: 68, 0.012±0.011
2: 8, 0.005±0.001
ms
0: 178, 207±25
1: 48, 203±22
2: 5, 217±27
Heart rate
n
bpm
0: 204, 262±34
1: 68, 273±33
2: 8, 308±32
bpm

hcn4l

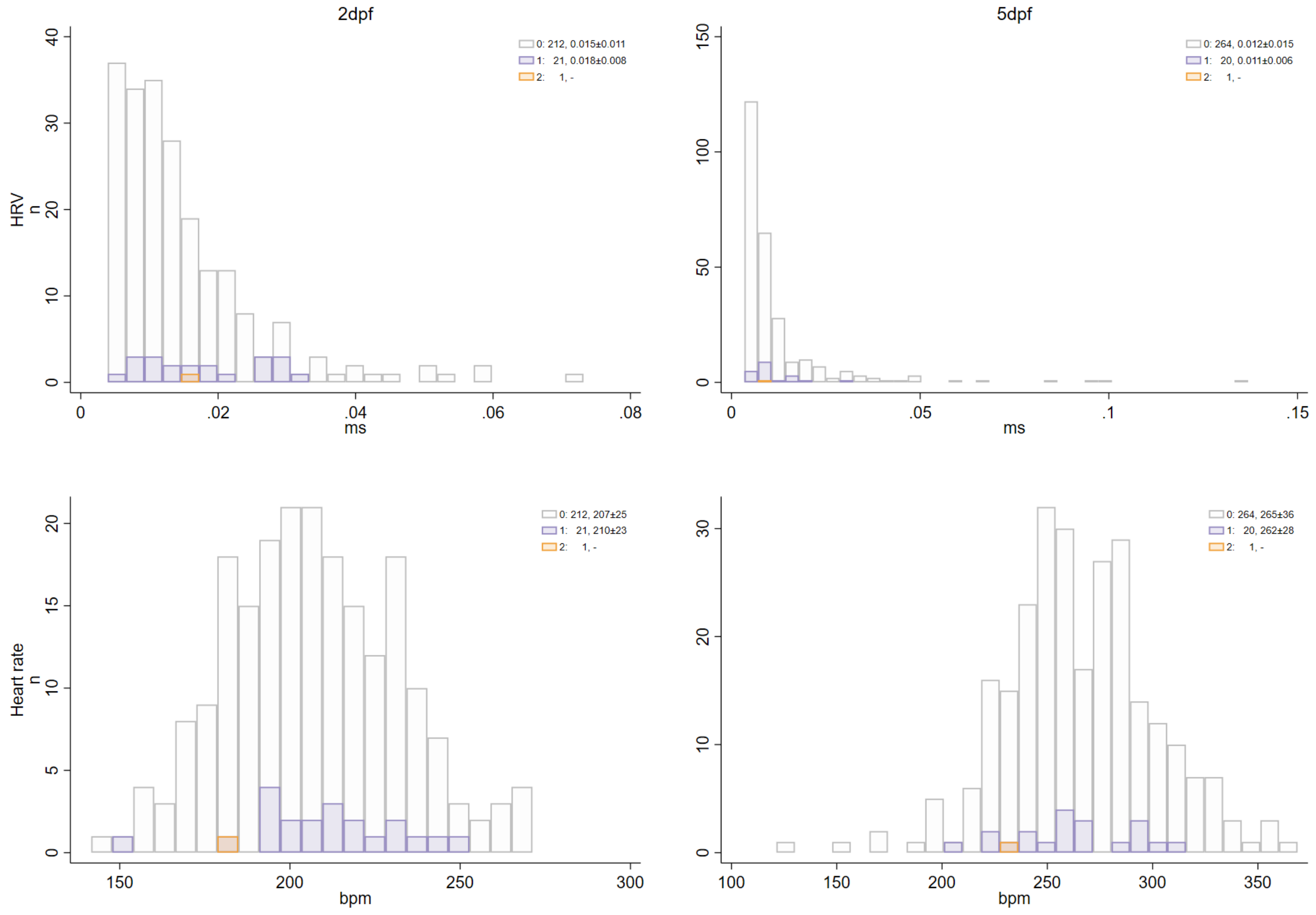
2dpf
0: 212, 0.015±0.011
1: 21, 0.018±0.008
2: 1, -
HRV
n
ms
5dpf
0: 264, 0.012±0.015
1: 20, 0.011±0.006
2: 1, -
ms
0: 212, 207±25
1: 21, 210±23
2: 1, -
Heart rate
n
bpm
0: 264, 265±36
1: 20, 262±28
2: 1, -
bpm

neo1a

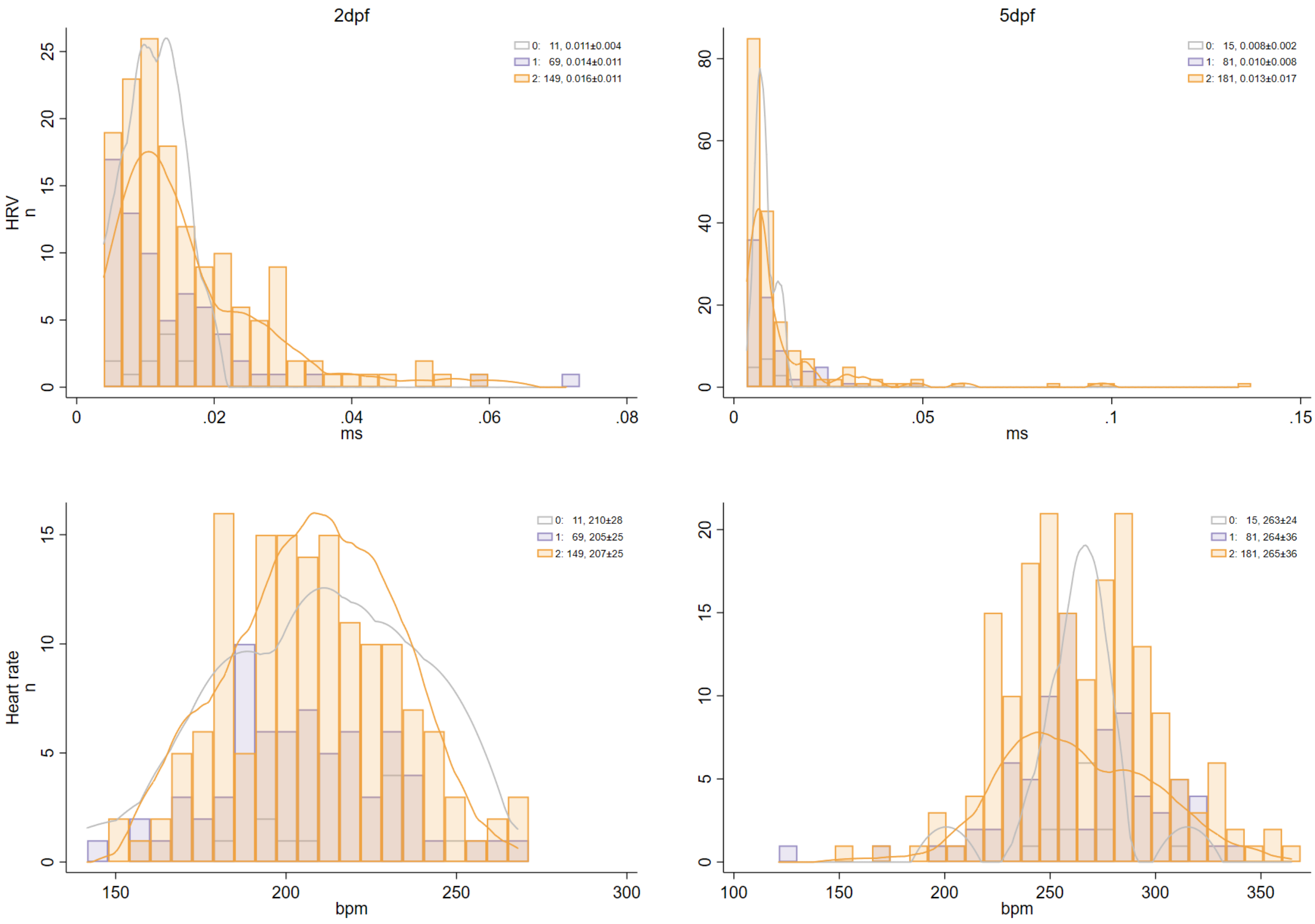
2dpf
5dpf
HRV
n
ms
0: 11, 0.011±0.004
1: 69, 0.014±0.011
2: 149, 0.016±0.011
0: 15, 0.008±0.002
1: 81, 0.010±0.008
2: 181, 0.013±0.017
Heart rate
n
bpm
0: 11, 210±28
1: 69, 205±25
2: 149, 207±25
0: 15, 263±24
1: 81, 264±36
2: 181, 265±36

neo1b

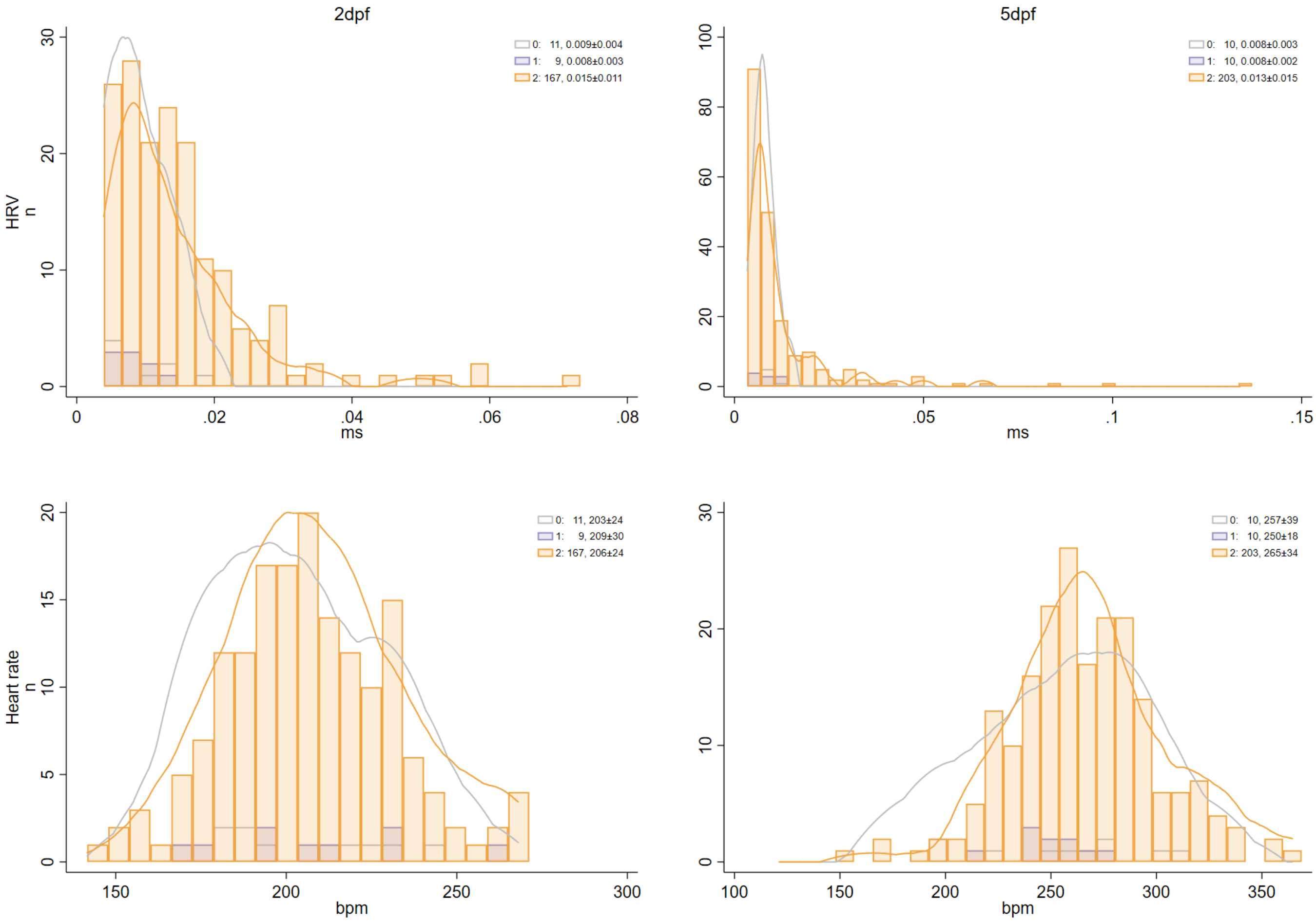
2dpf
5dpf
HRV
n
ms
0: 11, 0.009±0.004
1: 9, 0.008±0.003
2: 167, 0.015±0.011
0: 10, 0.008±0.003
1: 10, 0.008±0.002
2: 203, 0.013±0.015
Heart rate
n
bpm
0: 11, 203±24
1: 9, 209±30
2: 167, 206±24
0: 10, 257±39
1: 10, 250±18
2: 203, 265±34

quo (KIAA1755)

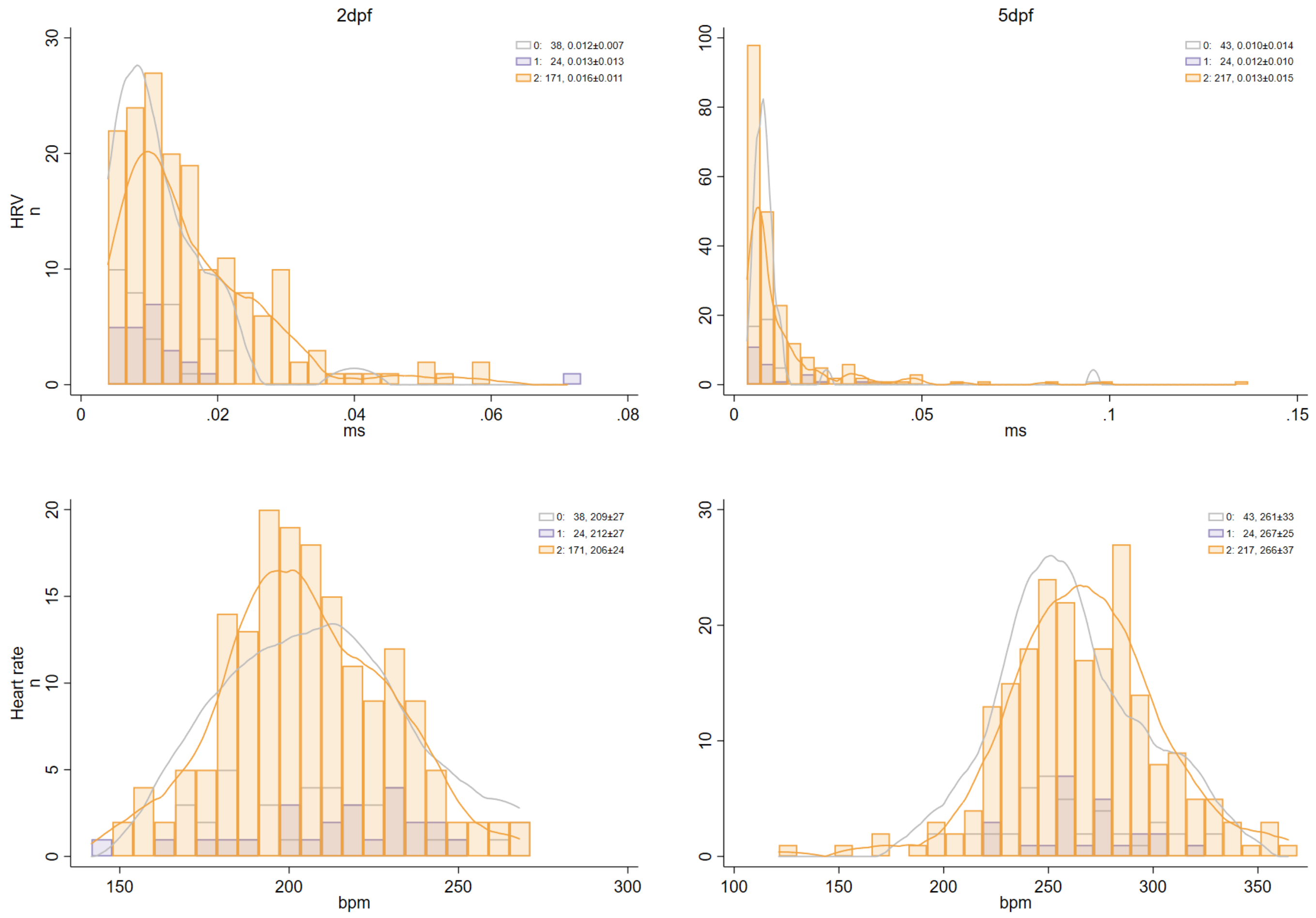

si:dkey-65j6.2 (KIAA1755)

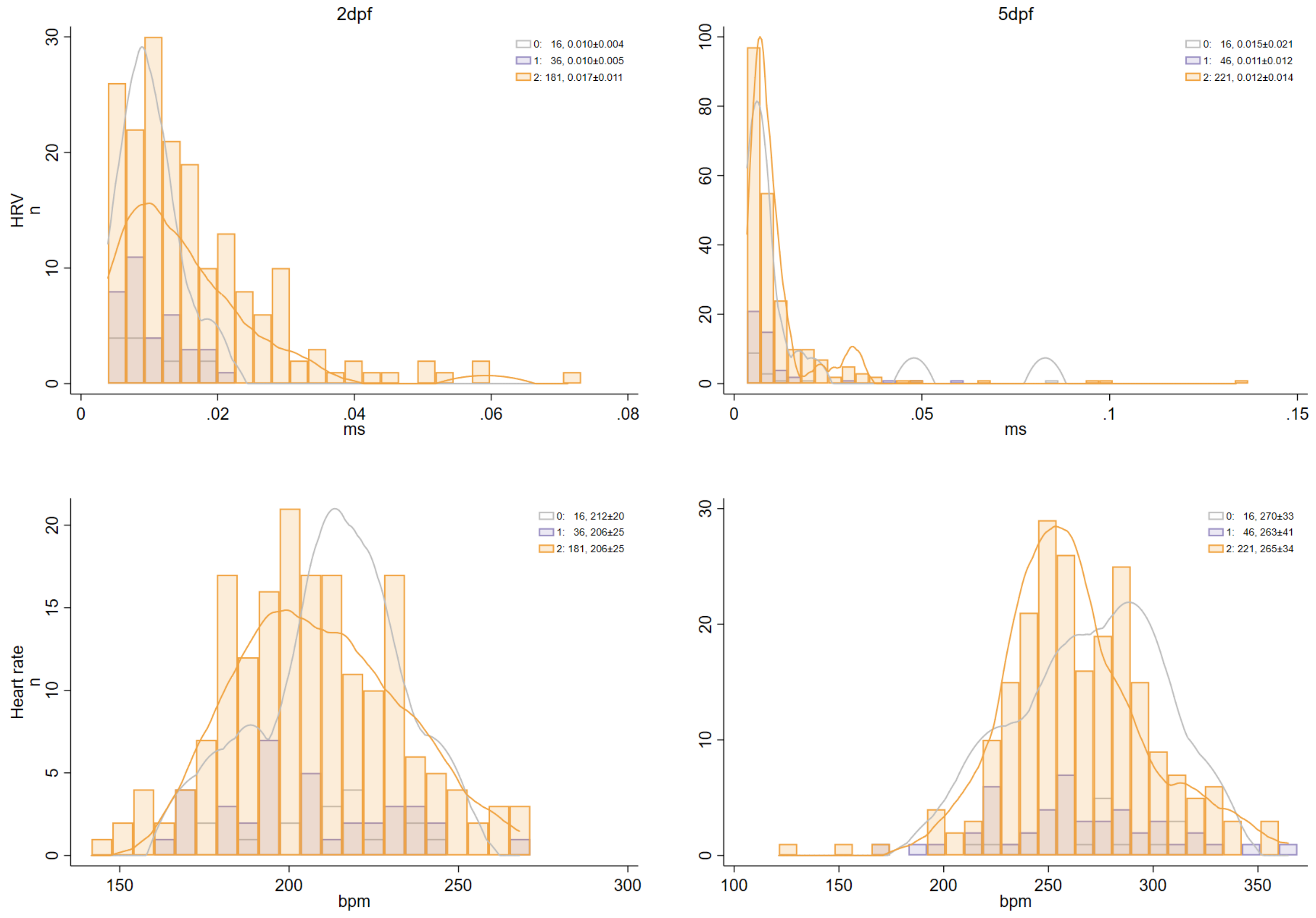

Supplementary Figure 3: Distributions of heart rate variability (HRV, in ms) and heart rate (in beats per min, bpm) shown in all embryos combined, as well as stratified by the number of mutated alleles for each of the nine CRISPR/Cas9 targeted candidate genes. In each histogram, the mean±SD in embryos with 0, 1 and 2 mutated alleles is shown in the top right corner. Orange and gray lines show Kernel density plots for embryos with CRISPR/Cas9-induced nonsense mutations in both alleles, and for embryos free from CRISPR/Cas9-induced mutations, respectively if n>5 for both.

Overall distribution

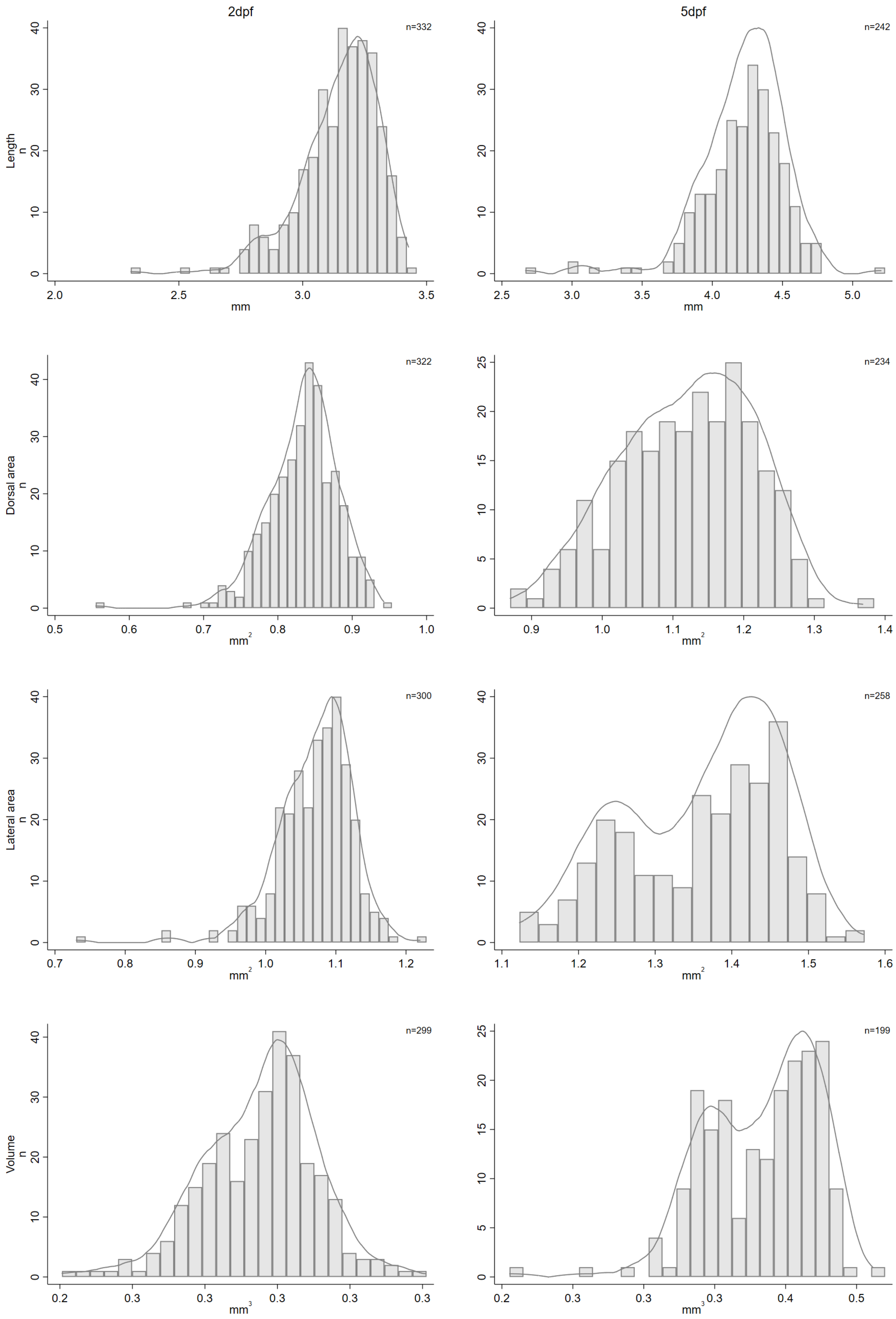

gngt1

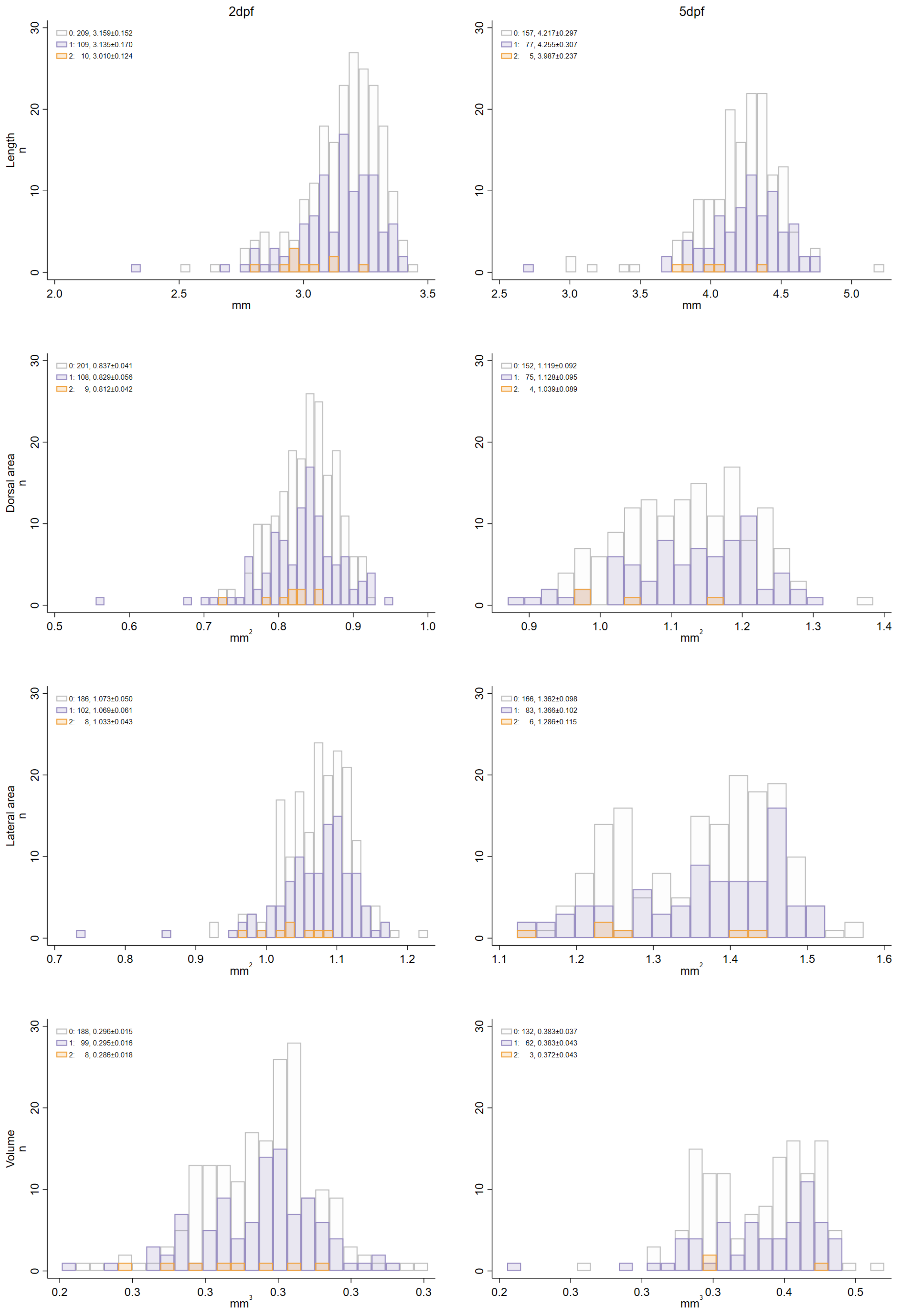
2dpf
5dpf
Length
n
Dorsal area
Lateral area
Volume
mm
mm²
mm³
0: 209, 3.159±0.152
1: 109, 3.135±0.170
2: 10, 3.010±0.124
0: 157, 4.217±0.297
1: 77, 4.255±0.307
2: 5, 3.987±0.237
0: 201, 0.837±0.041
1: 108, 0.829±0.056
2: 9, 0.812±0.042
0: 152, 1.119±0.092
1: 75, 1.128±0.095
2: 4, 1.039±0.089
0: 186, 1.073±0.050
1: 102, 1.069±0.061
2: 8, 1.033±0.043
0: 166, 1.362±0.098
1: 83, 1.366±0.102
2: 6, 1.286±0.115
0: 188, 0.296±0.015
1: 99, 0.295±0.016
2: 8, 0.286±0.018
0: 132, 0.383±0.037
1: 62, 0.383±0.043
2: 3, 0.372±0.043

syt10

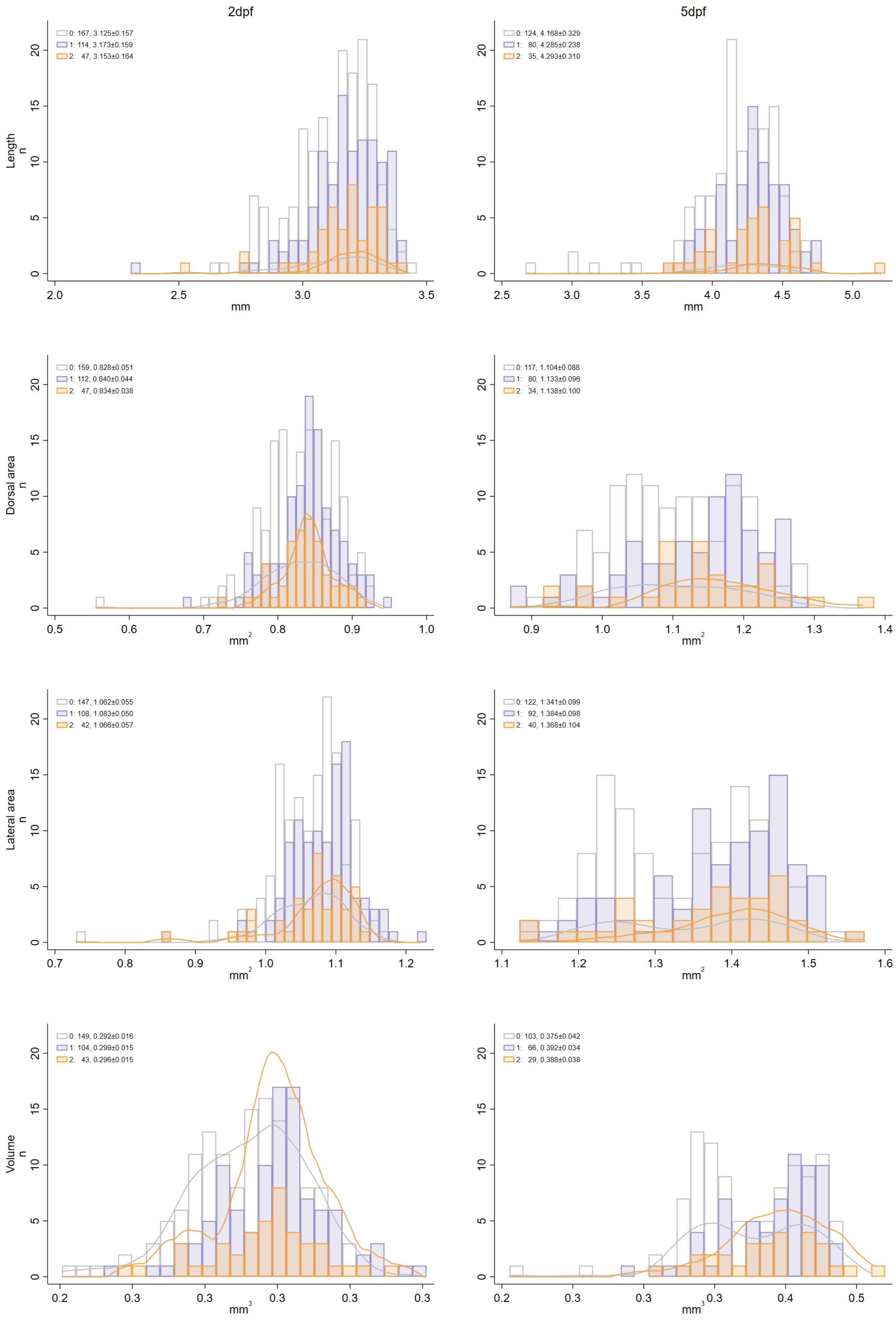
2dpf
5dpf
Length
n
0: 167, 3.125±0.157
1: 114, 3.173±0.159
2: 47, 3.153±0.164
mm
0: 124, 4.168±0.329
1: 80, 4.285±0.238
2: 35, 4.293±0.310
mm
Dorsal area
n
0: 159, 0.828±0.051
1: 112, 0.840±0.044
2: 47, 0.834±0.038
mm²
0: 117, 1.104±0.088
1: 80, 1.133±0.096
2: 34, 1.138±0.100
mm²
Lateral area
n
0: 147, 1.062±0.055
1: 108, 1.083±0.050
2: 42, 1.066±0.057
mm²
0: 122, 1.341±0.099
1: 92, 1.384±0.098
2: 40, 1.368±0.104
mm²
Volume
n
0: 149, 0.292±0.016
1: 104, 0.299±0.015
2: 43, 0.296±0.015
mm³
0: 103, 0.375±0.042
1: 66, 0.392±0.034
2: 29, 0.388±0.038
mm³

rgs6

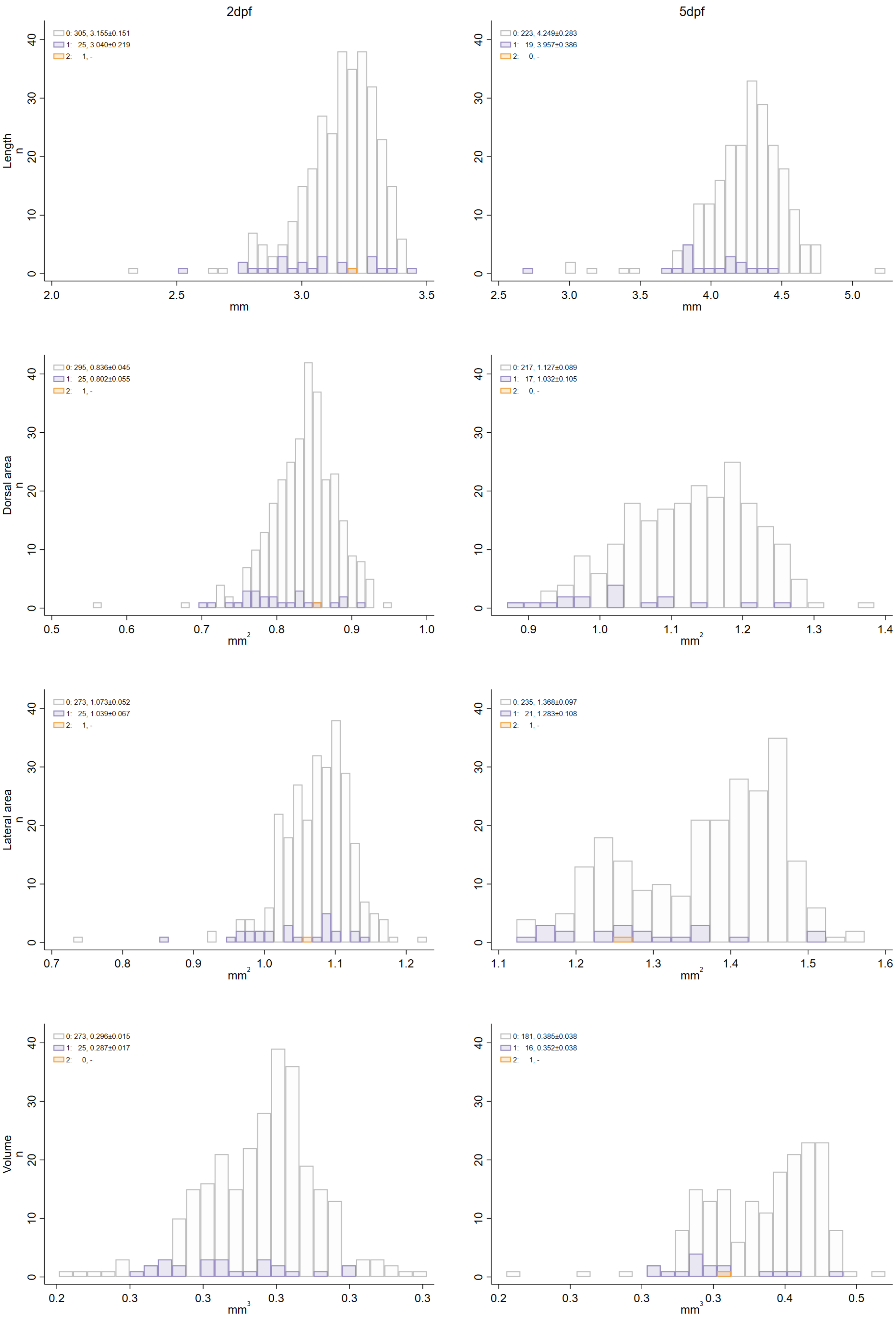

2dpf
5dpf
Length
Dorsal area
Lateral area
Volume
n
mm
mm²
mm³
0: 305, 3.155±0.151
1: 25, 3.040±0.219
2: 1, -
0: 223, 4.249±0.283
1: 19, 3.957±0.386
2: 0, -
0: 295, 0.836±0.045
1: 25, 0.802±0.055
2: 1, -
0: 217, 1.127±0.089
1: 17, 1.032±0.105
2: 0, -
0: 273, 1.073±0.052
1: 25, 1.039±0.067
2: 1, -
0: 235, 1.368±0.097
1: 21, 1.283±0.108
2: 1, -
0: 273, 0.296±0.015
1: 25, 0.287±0.017
2: 0, -
0: 181, 0.385±0.038
1: 16, 0.352±0.038
2: 1, -

hcn4

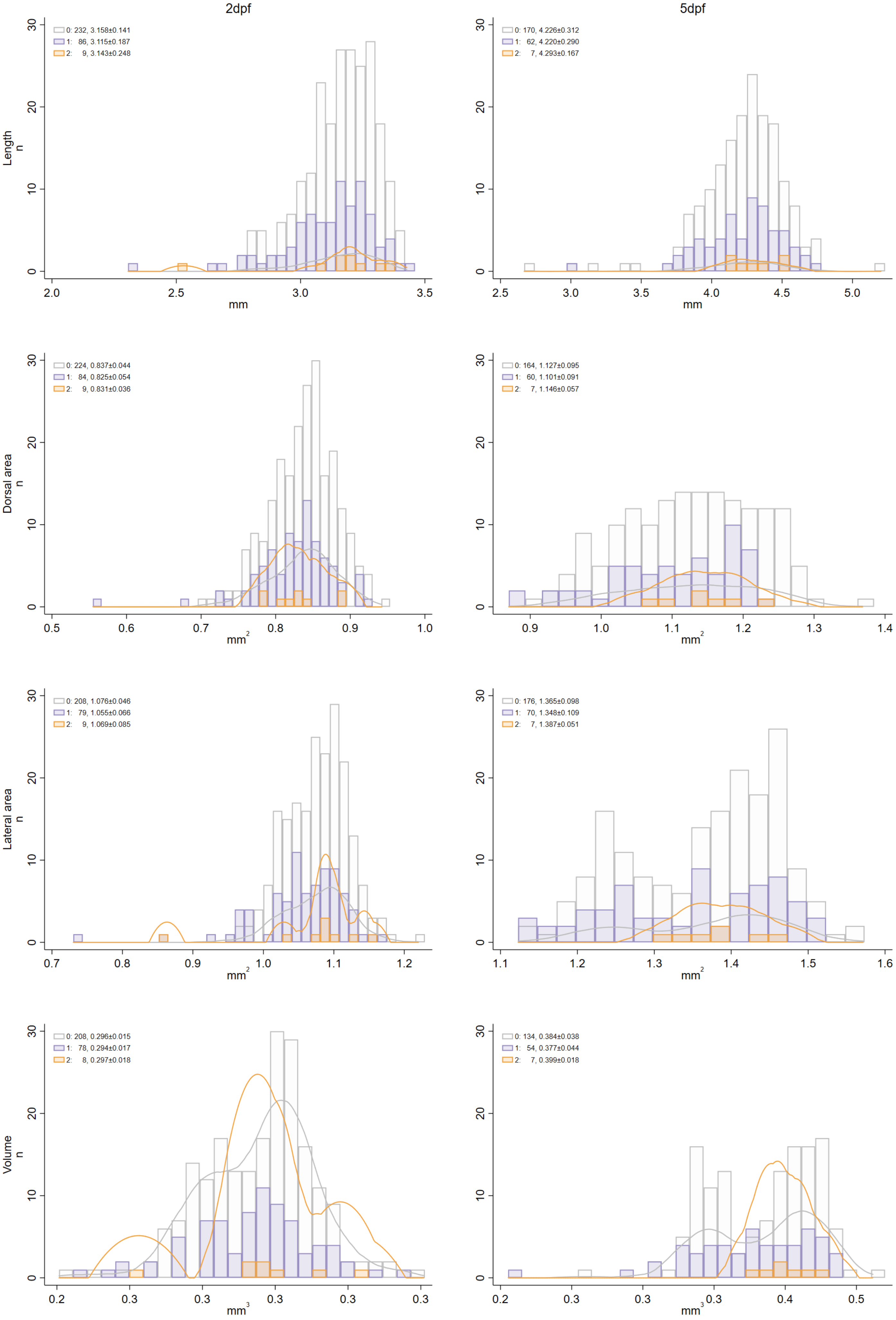
2dpf
5dpf
Length
n
Dorsal area
Lateral area
Volume
mm
mm2
mm3
0: 232, 3.158±0.141
1: 86, 3.115±0.187
2: 9, 3.143±0.248
0: 170, 4.226±0.312
1: 62, 4.220±0.290
2: 7, 4.293±0.167
0: 224, 0.837±0.044
1: 84, 0.825±0.054
2: 9, 0.831±0.036
0: 164, 1.127±0.095
1: 60, 1.101±0.091
2: 7, 1.146±0.057
0: 208, 1.076±0.046
1: 79, 1.055±0.066
2: 9, 1.069±0.085
0: 176, 1.365±0.098
1: 70, 1.348±0.109
2: 7, 1.387±0.051
0: 208, 0.296±0.015
1: 78, 0.294±0.017
2: 8, 0.297±0.018
0: 134, 0.384±0.038
1: 54, 0.377±0.044
2: 7, 0.399±0.018

hcn4l

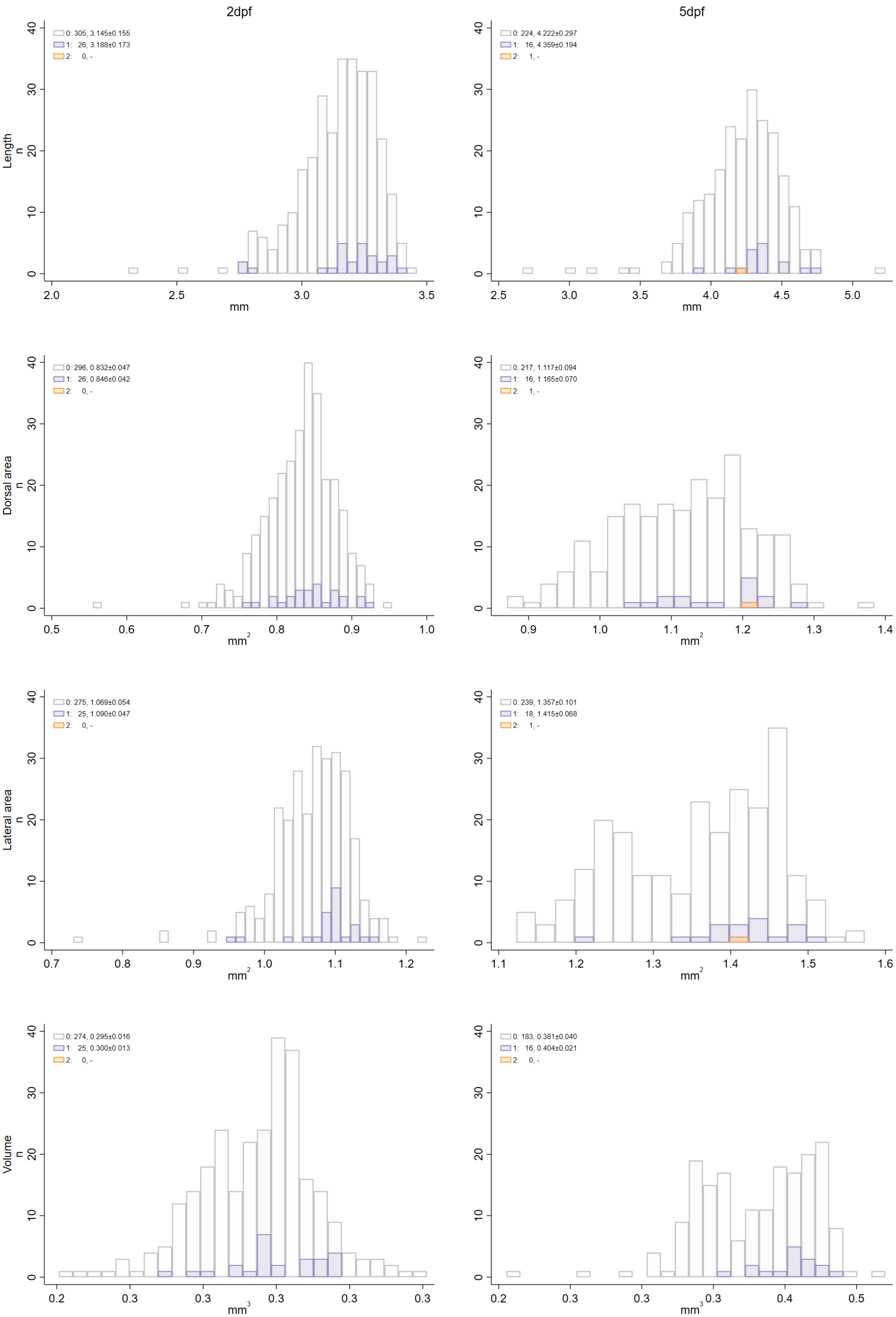
2dpf
5dpf
Length
Dorsal area
Lateral area
Volume
n
0: 305, 3.145±0.155
1: 26, 3.188±0.173
2: 0, -
0: 224, 4.222±0.297
1: 16, 4.359±0.194
2: 1, -
0: 296, 0.832±0.047
1: 26, 0.846±0.042
2: 0, -
0: 217, 1.117±0.094
1: 16, 1.165±0.070
2: 1, -
0: 275, 1.069±0.054
1: 25, 1.090±0.047
2: 0, -
0: 239, 1.357±0.101
1: 18, 1.415±0.068
2: 1, -
0: 274, 0.295±0.016
1: 25, 0.300±0.013
2: 0, -
0: 183, 0.381±0.040
1: 16, 0.404±0.021
2: 0, -
mm
mm²
mm³

neo1a

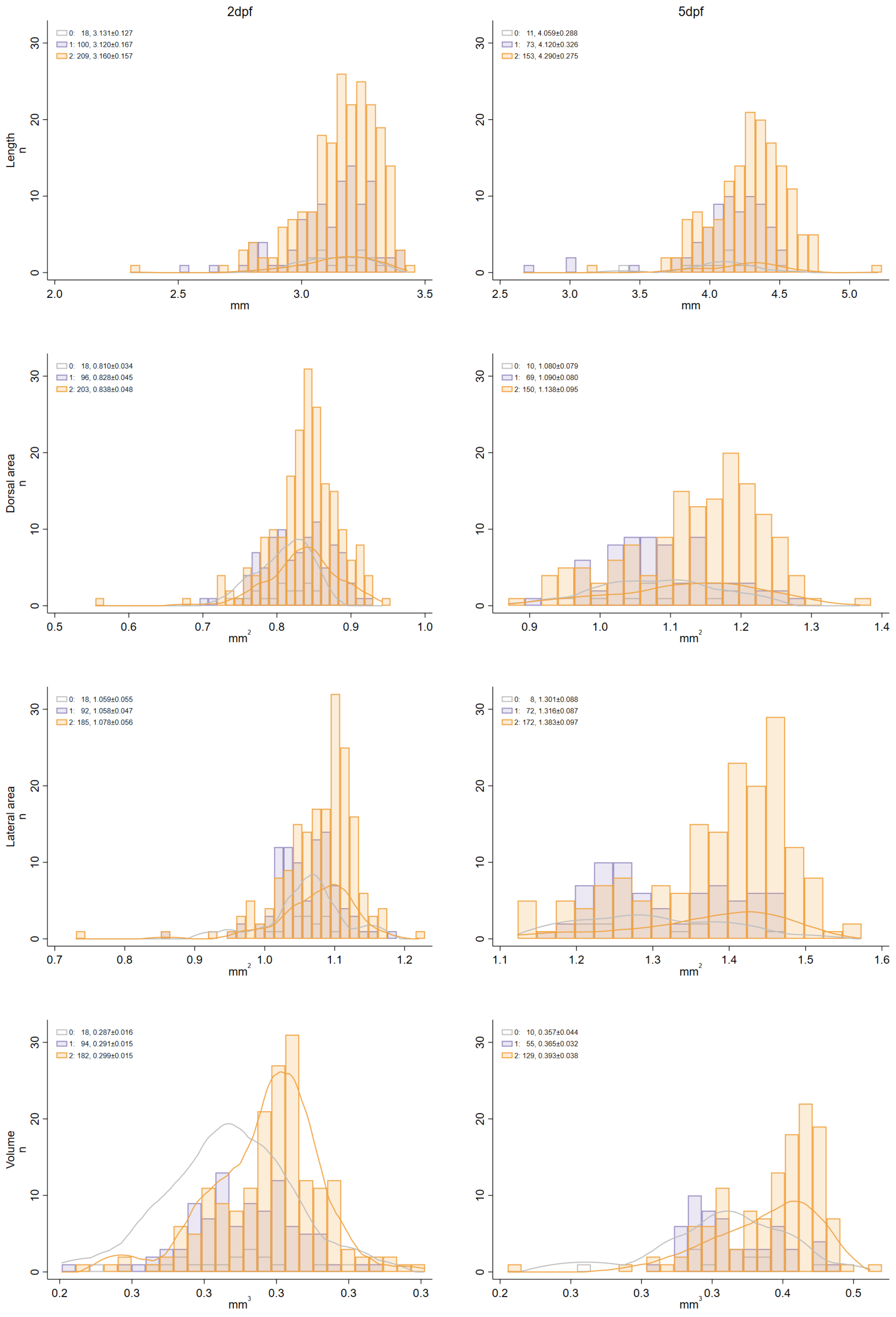
2dpf
5dpf
Length
Dorsal area
Lateral area
Volume
n
mm
mm²
mm³
0: 18, 3.131±0.127
1: 100, 3.120±0.167
2: 209, 3.160±0.157
0: 11, 4.059±0.288
1: 73, 4.120±0.326
2: 153, 4.290±0.275
0: 18, 0.810±0.034
1: 96, 0.828±0.045
2: 203, 0.838±0.048
0: 10, 1.080±0.079
1: 69, 1.090±0.080
2: 150, 1.138±0.095
0: 18, 1.059±0.055
1: 92, 1.058±0.047
2: 185, 1.078±0.056
0: 8, 1.301±0.088
1: 72, 1.316±0.087
2: 172, 1.383±0.097
0: 18, 0.287±0.016
1: 94, 0.291±0.015
2: 182, 0.299±0.015
0: 10, 0.357±0.044
1: 55, 0.365±0.032
2: 129, 0.393±0.038

neo1b

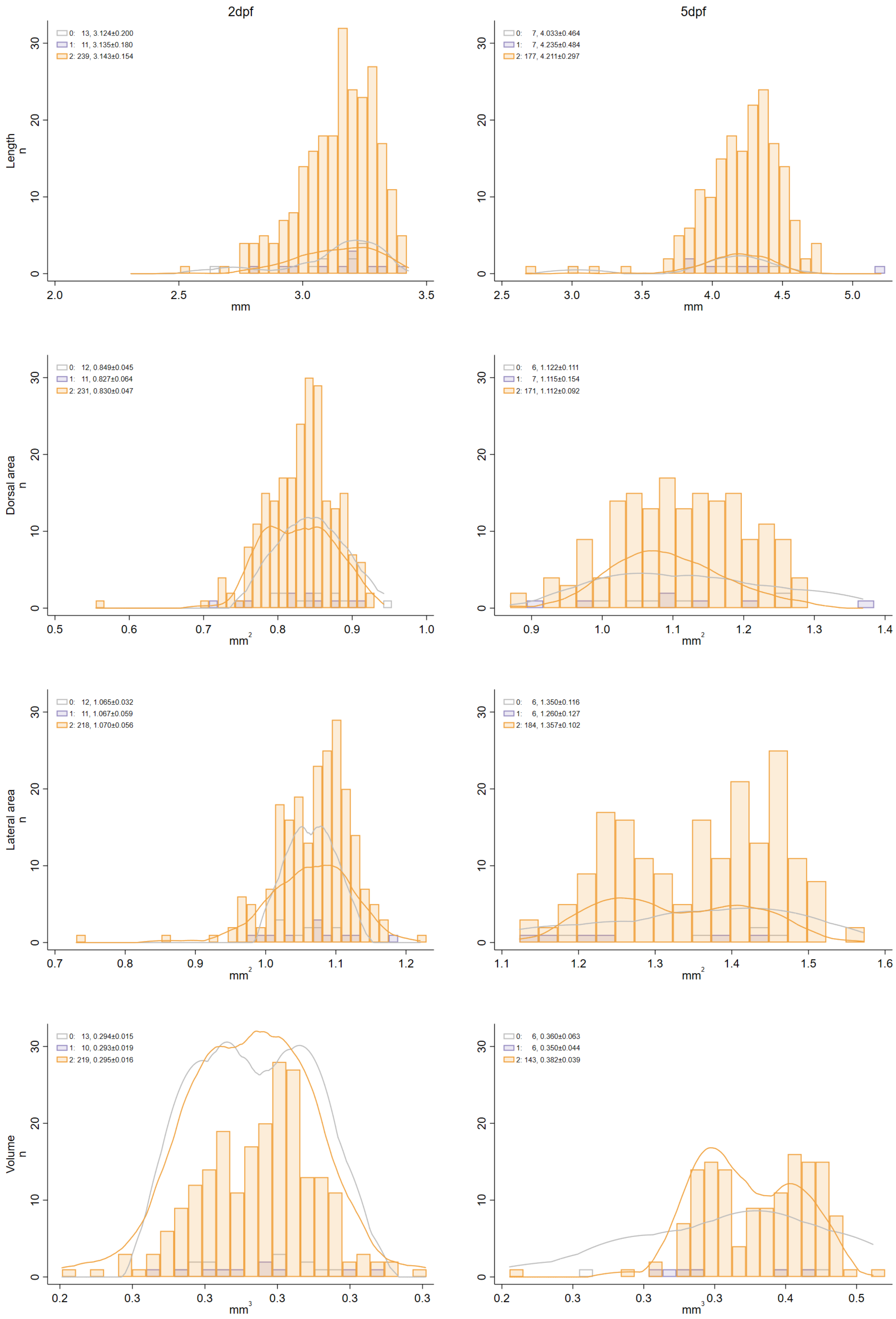
2dpf
5dpf
Length
Dorsal area
Lateral area
Volume
n
mm
mm²
mm³
0: 13, 3.124±0.200
1: 11, 3.135±0.180
2: 239, 3.143±0.154
0: 7, 4.033±0.464
1: 7, 4.235±0.484
2: 177, 4.211±0.297
0: 12, 0.849±0.045
1: 11, 0.827±0.064
2: 231, 0.830±0.047
0: 6, 1.122±0.111
1: 7, 1.115±0.154
2: 171, 1.112±0.092
0: 12, 1.065±0.032
1: 11, 1.067±0.059
2: 218, 1.070±0.056
0: 6, 1.350±0.116
1: 6, 1.260±0.127
2: 184, 1.357±0.102
0: 13, 0.294±0.015
1: 10, 0.293±0.019
2: 219, 0.295±0.016
0: 6, 0.360±0.063
1: 6, 0.350±0.044
2: 143, 0.382±0.039

quo (KIAA1755)

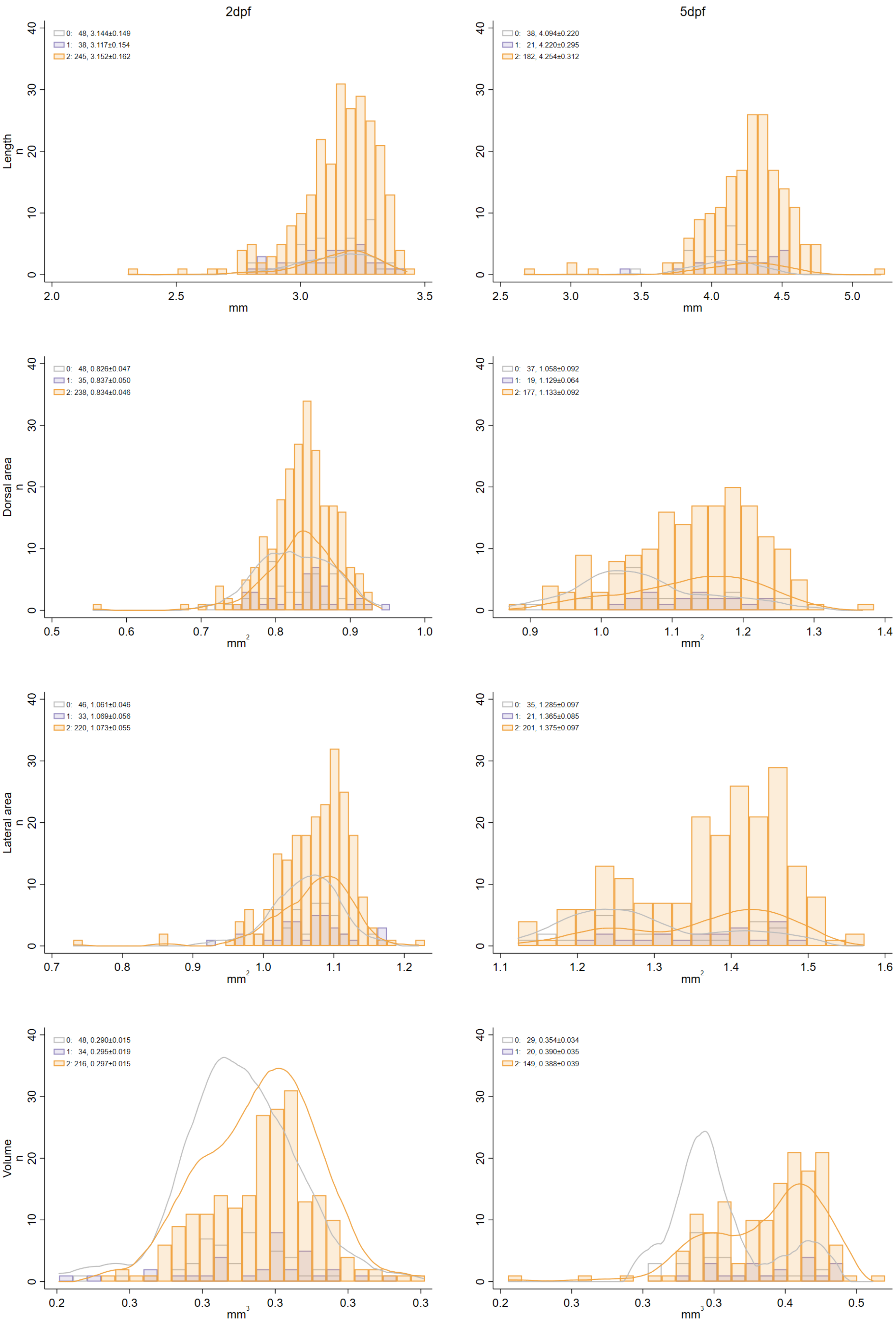

si:dkey-65j6.2 (KIAA1755)

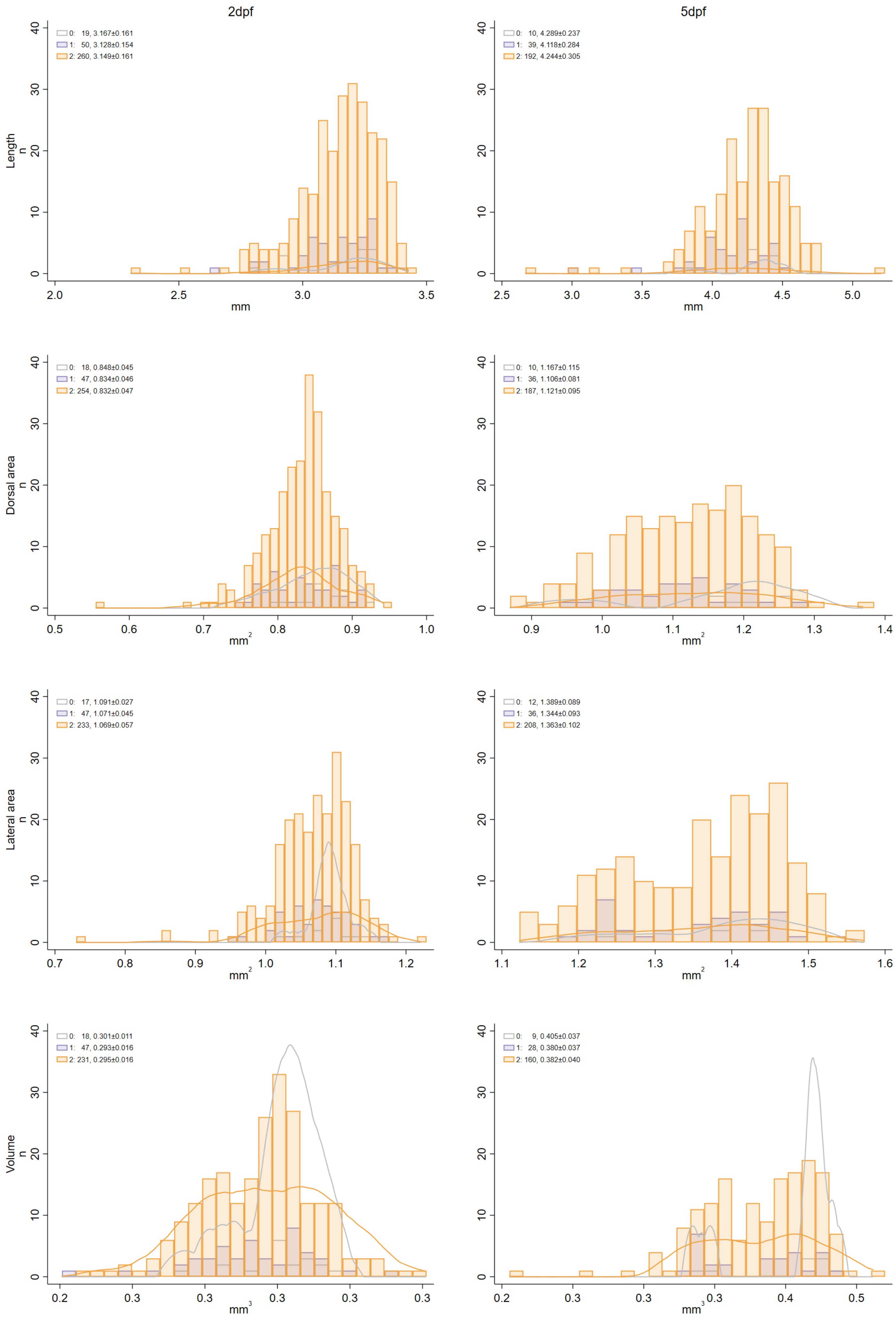

Supplementary Figure 4: Distributions of body length, dorsal and lateral body surface area, and body volume shown in all embryos combined, as well as stratified by the number of mutated alleles for each of the nine CRISPR/Cas9 targeted candidate genes. In each histogram, the mean±SD in embryos with 0, 1 and 2 mutated alleles is shown in the top left corner. Orange and gray lines show Kernel density plots for embryos with CRISPR/Cas9-induced nonsense mutations in both alleles, and for embryos free from CRISPR/Cas9-induced mutations, respectively, if n>5 for both.

gngt1

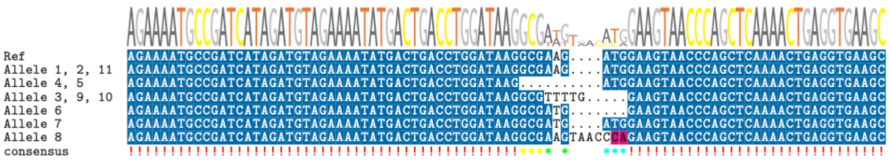

	n	Mean	SD
Allele 1, 2, 11	566	505	377
Allele 4, 5	75	514	384
Allele 3, 9, 10	69	529	409
Allele 6	3	606	119
Allele 7	3	504	221
Allele 8	2	526	143

syt10

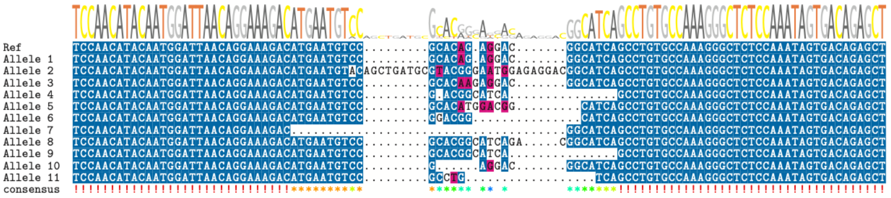

	n	Mean	SD
Allele 1	325	1024	644
Allele 2	44	735	462
Allele 3	42	793	405
Allele 4	42	368	246
Allele 5	40	606	397
Allele 6	19	667	267
Allele 7	15	963	431
Allele 8	11	937	645
Allele 9	11	502	300
Allele 10	4	524	229
Allele 11	3	1247	670

rgs6

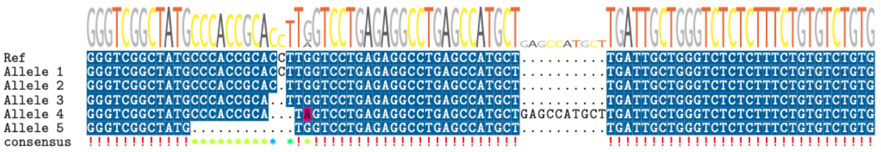

	n	Mean	SD
Allele 1	381	1431	813
Allele 2	24	784	453
Allele 3	7	540	195
Allele 4	4	920	287
Allele 5	1	1424	

hcn4

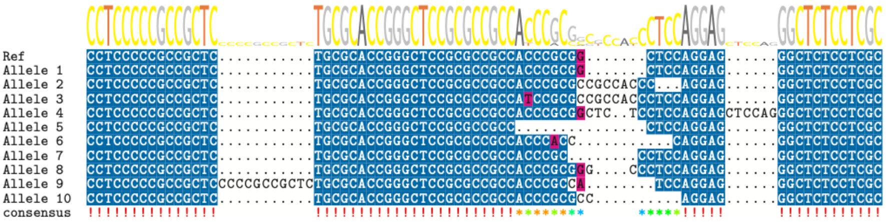

	n	Mean	SD
Allele 1	369	1380	789
Allele 2	48	725	343
Allele 3	16	1314	225
Allele 4	15	799	383
Allele 5	13	917	563
Allele 6	11	987	377
Allele 7	9	1302	587
Allele 8	2	694	49
Allele 9	2	346	36
Allele 10	1	222	

hcn4l

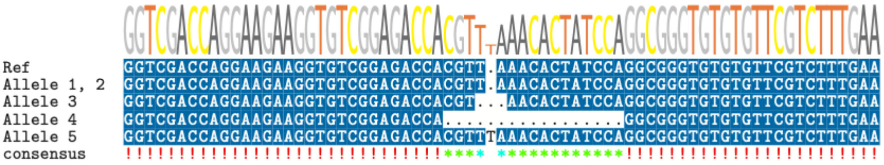

	n	Mean	SD
Allele 1, 2	418	1048	657
Allele 3	18	585	295
Allele 4	9	523	176
Allele 5	4	1218	915

	n	Mean	SD
Allele 1	132	941	691
Allele 2	89	957	545
Allele 3	82	899	475
Allele 4	69	589	305
Allele 5	68	696	413
Allele 6	66	703	359
Allele 7	57	985	521
Allele 8	20	960	653
Allele 9	20	633	415
Allele 10	17	1026	474
Allele 11	16	672	404
Allele 12	14	688	336
Allele 13	11	807	252
Allele 14	10	569	314
Allele 15	9	1179	312
Allele 16	6	760	543
Allele 17	5	1569	1406
Allele 18	5	1451	663
Allele 19	5	1010	552
Allele 20	5	754	361
Allele 21	2	734	441
Allele 22	2	376	40
Allele 23	1	1242	
Allele 24	1	698	

neo1a

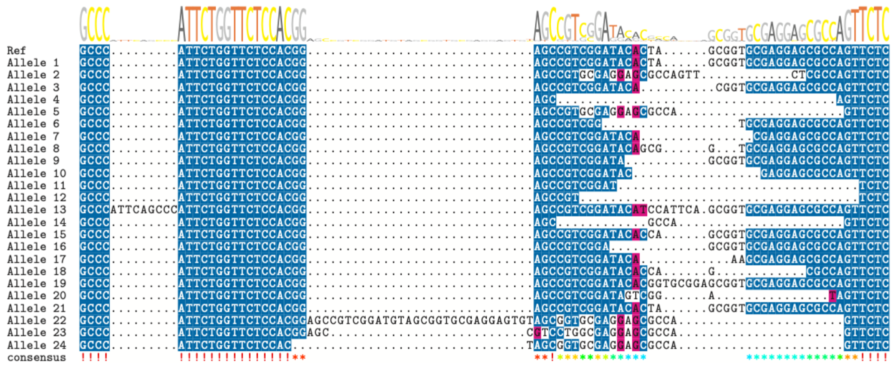

neo1b

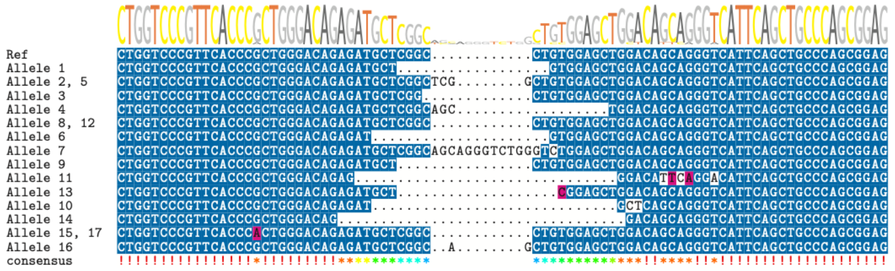

	n	Mean	SD
Allele 1	102	733	617
Allele 2, 5	68	985	792
Allele 3	58	632	483
Allele 4	50	700	505
Allele 8, 12	35	393	421
Allele 6	19	739	686
Allele 7	15	915	914
Allele 9	13	689	469
Allele 11	7	801	567
Allele 13	7	503	266
Allele 10	6	960	710
Allele 14	2	1170	685
Allele 15, 17	2	4	2
Allele 16	1	5	

quo

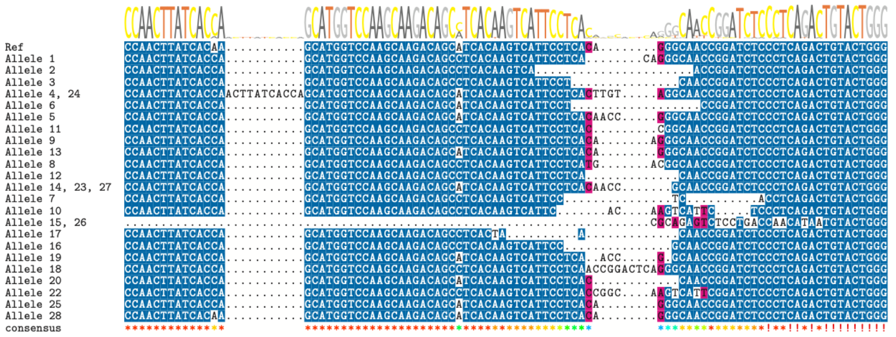

	n	Mean	SD
Allele 1	77	997	507
Allele 2	70	818	888
Allele 3	61	1117	569
Allele 4, 24	53	1175	598
Allele 6	51	541	417
Allele 5	49	780	596
Allele 11	24	593	552
Allele 9	23	1077	490
Allele 13	22	876	459
Allele 8	21	775	588
Allele 12	17	1190	408
Allele 14, 23, 27	15	1469	683
Allele 7	14	1365	561
Allele 10	13	1602	834
Allele 15, 26	13	1005	591
Allele 17	10	876	386
Allele 16	9	1063	794
Allele 19	9	618	283
Allele 18	7	950	426
Allele 20	6	581	250
Allele 21	4	1101	323
Allele 22	4	120	48
Allele 25	1	1184	
Allele 28	1	25	

si:dkey-65j6.2

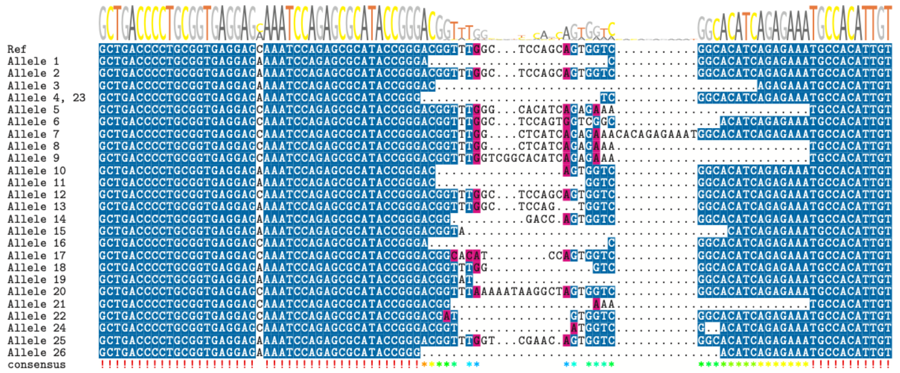

	n	Mean	SD
Allele 1	92	985	638
Allele 2	63	951	613
Allele 4, 23	62	887	460
Allele 5	57	856	414
Allele 6	51	772	333
Allele 3	50	1070	464
Allele 7	41	829	457
Allele 8	34	935	587
Allele 9	32	837	470
Allele 12	27	564	351
Allele 10	21	964	370
Allele 13	18	793	426
Allele 14	18	787	595
Allele 11	15	1152	331
Allele 18	13	657	443
Allele 15	12	1035	370
Allele 16	9	1110	532
Allele 17	9	970	382
Allele 20	7	584	214
Allele 22	4	726	358
Allele 19	3	1411	177
Allele 21	3	976	130
Allele 24	3	834	128
Allele 25	1	562	
Allele 26	1	558	

Supplementary Figure 5: Unique alleles in nine candidate genes showing CRISPR/Cas9-induced mutations compared with the zebrafish reference genome, GRCz11. Colour coding illustrates if base pairs are ≥50% conserved (blue), similar (pink) or non-conserved (white). Tables on the right show the number times each allele appears across the 381 successfully sequenced embryos (max 2x381), and the mean and standard deviation for the number of reads calls are based on. Alleles that differ by inherently present variants not attributed to CRISPR/Cas9 are grouped together as indicated in the figure.

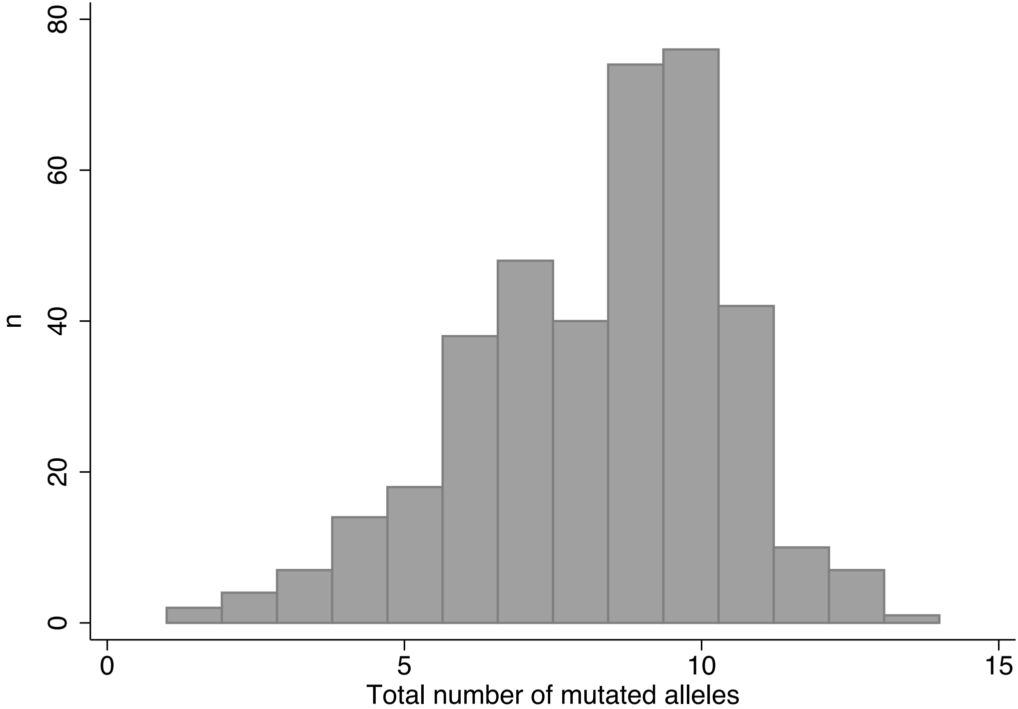

Supplementary Figure 6: Distribution of the number of mutated alleles across the nine CRISPR-targeted sites. A mutation is defined as any previously undescribed variant located within ±30bp of the CRISPR/Cas9 cut site.

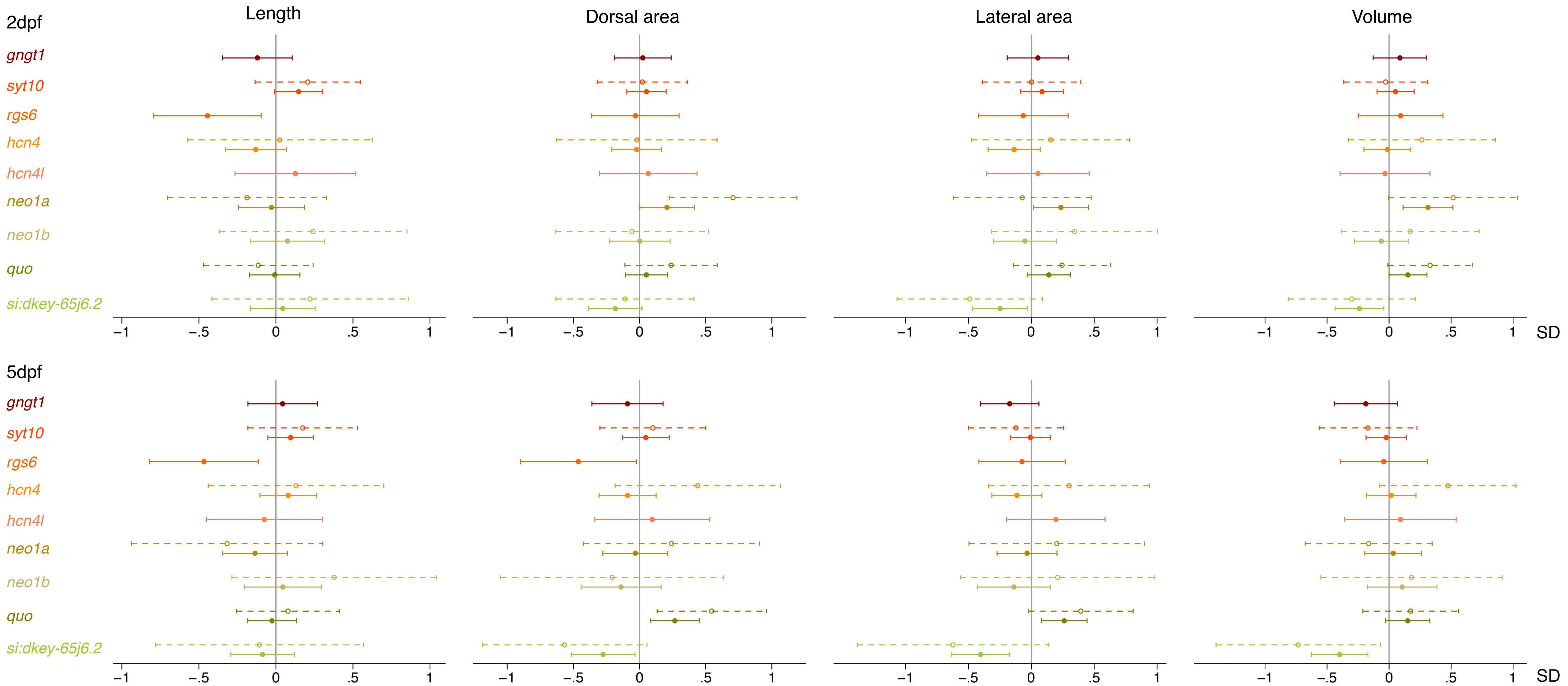

Supplementary Figure 7: Effect of mutations in candidate genes on body length; and on dorsal body surface area, lateral body surface area and body volume normalised for length at 2 days post-fertilization (dpf, top) and 5dpf (bottom). Fulldots and solid whiskers show the effect size and 95% confidence interval for each additional mutated allele, weighted by the predicted effect on protein function. Open dots and dotted whiskers indicate the effect size and 95% confidence interval for frameshift or premature stop codon inducing mutations in two alleles vs. no CRISPR-induced mutations. Effects were adjusted for the weighted number of mutated alleles in the other genes, as well as for time of day (fixed factors), with embryos nested in batches (random factor). *quo* and *si:dkey-65j6.2* are orthologues of the human *KIAA1755*.

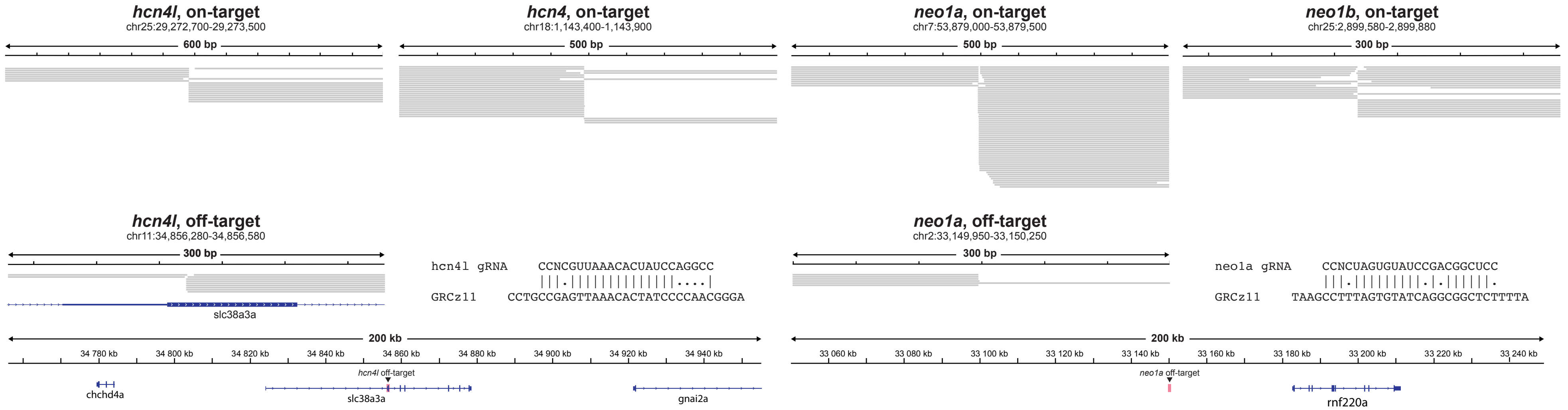

Supplementary Figure 8: *In vitro* Nano off-target sequencing results for the *hcn4*, *hcn4l*, *neo1a* and *neo1b* gRNAs in DNA extracted from a Tg(*acta2*:GFP) positive fish used to generate CRISPR/Cas9 founders. One off-target site was observed for the *hcn4l* and *neo1a* gRNAs, in spite of five and four mismatches, respectively. For *hcn4l,* this off-target site was in the coding region of *slc38a3a*. Off-target mutagenic activity was lower than on-target activity for both gRNAs.

Ivabradine treatment

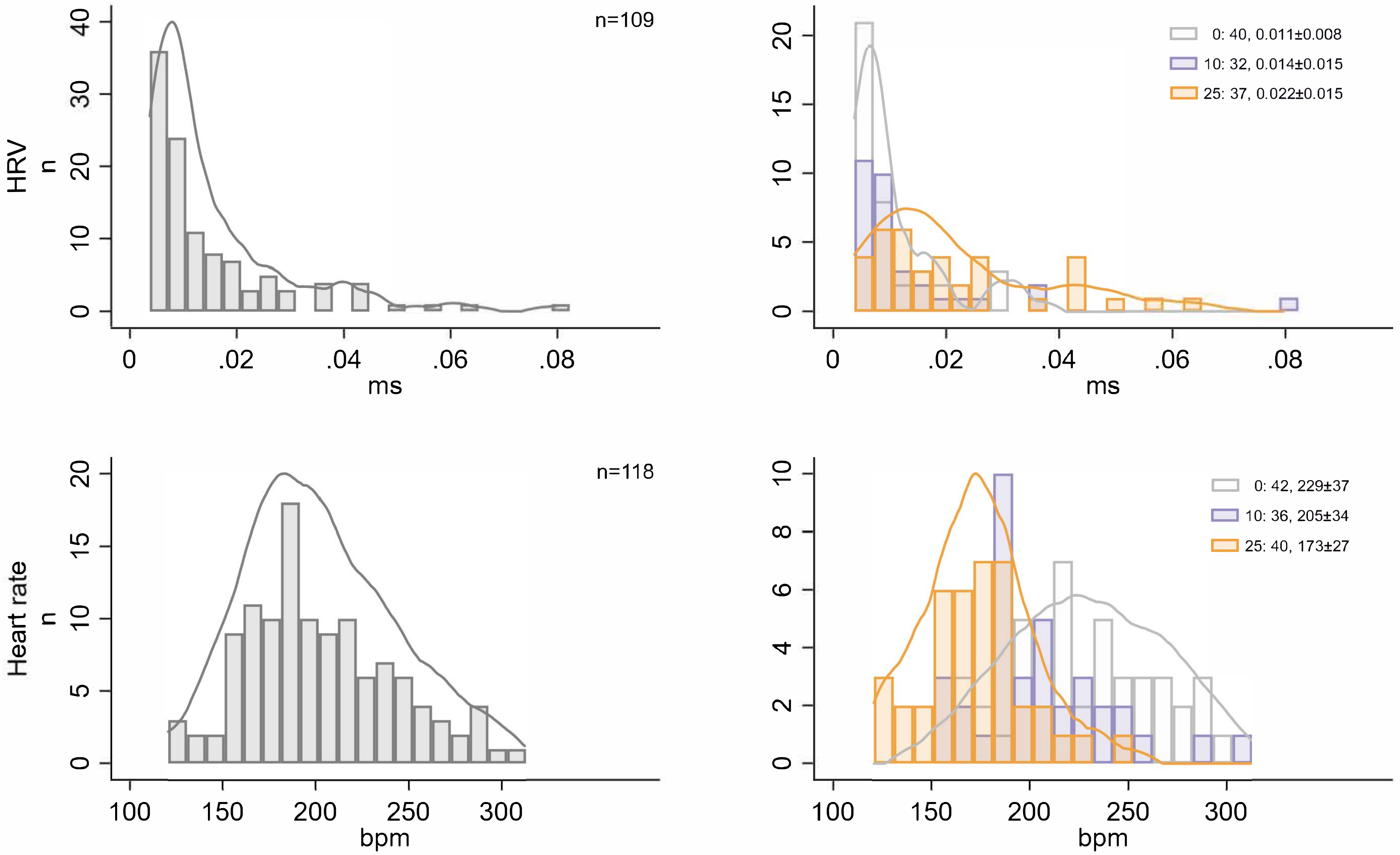

Supplementary Figure 9: Distributions of heart rate variability (HRV, in ms) and heart rate (in beats per min, bpm) shown in all embryos combined (left), as well as stratified by ivabradine treatment condition (right). In each histogram, the mean±SD in embryos treated with 0, 10 and 25μM ivabradine is shown in the top right corner. Orange and gray lines show Kernel density plots for embryos treated with 25 and 0 μM ivabradine, respectively.

SUPPLEMENTARY TABLES

Supplementary Table 1 – Overview of zebrafish orthologues of human candidate genes, CRISPR/Cas9 guide RNAs, and predicted off-targets (page 31)

Supplementary Table 2 – Overview of CRISPR/Cas9 guide RNAs (page 32)

Supplementary Table 3 – Unique variants and affected transcripts for each targeted zebrafish orthologue (page 33-37)

Supplementary Table 4 – Mutant allele frequencies for CRISPR/Cas9-induced mutations by zebrafish orthologue (page 38)

Supplementary Table 5 – Additive effects of CRISPR/Cas9-induced mutations on sinoatrial pauses and arrests (page 39)

Supplementary Table 6 – Effects of CRISPR/Cas9-induced nonsense mutations in both alleles vs. no CRISPR/Cas9-induced mutations on sinoatrial pauses and arrests at 2dpf (page 40)

Supplementary Table 7 – Additive effects of CRISPR/Cas9-induced mutations on (change in) heart rate variability and heart rate (page 41)

Supplementary Table 8 – Effects of CRISPR/Cas9-induced nonsense mutations in both alleles vs. no CRISPR/Cas9-induced mutations on (change in) heart rate variability and heart rate (page 42)

Supplementary Table 9 – Additive effect of CRISPR/Cas9-induced mutations on body size (page 43)

Supplementary Table 10 – Effect of CRISPR/Cas9-induced nonsense mutations in both alleles vs. no CRISPR/Cas9-induced mutations on body size (page 44)

Supplementary Table 11 – Transcripts with at least 75% sequence similarity to the zebrafish hcn4 cDNA sequence (page 45)

Supplementary Table 12 – qRT-PCR target sites, primers and experimental conditions (page 46)

Supplementary Table 13 – Effects of targeting *hcn4*, *hcn4l*, or *hcn4* and *hcn4l* using CRISPR/Cas9 on the expression of genes with >75% sequence similarity to the main zebrafish hcn4 transcript (page 47)

Supplementary Table 14 – Druggability of interacting partners of putative causal genes (page 48)

Supplementary Table 15 – Effect of 24h of ivabradine treatment on heart rate variability and heart rate in 5dpf zebrafish embryos (page 49)

Supplementary Table 1: Overview of zebrafish orthologues of human candidate genes, CRISPR/Cas9 guide RNAs, and predicted off-targets

Human gene	ENSG Stable ID	Zebrafish orthologue	ENSDARG stable ID	Target %id	Query %id	Conservation Zebrafish - Human	Conservation Zebrafish - Spotted gar	Main zebrafish transcript
GNGT1 / GNG11	ENSG00000127928 / ENSG00000127920	*gngt1*	ENSDARG00000035798	67.12 / 61.64	66.22 / 61.64	17	21	ENSDART00000051950
SYT10	ENSG00000110975	*syt10*	ENSDARG00000045750	64.55	75.53	0	1	ENSDART00000136049
RGS6		*rgs6*	ENSDARG00000015627	82.21	82.04	9	20	ENSDART00000135513
HCN4	ENSG00000138622	*hcn4*	ENSDARG00000061685	63.1	58.27	6	3	ENSDART00000136140
HCN4	ENSG00000138622	*hcn4l*	ENSDARG00000074419	59.76	49.88	3	3	ENSDART00000088249
NEO1	ENSG00000067141	*neo1a*	ENSDARG00000102855	70.45	68.86	1	5	ENSDART00000158160
NEO1	ENSG00000067141	*neo1b*	ENSDARG00000075100	64.26	62.15	2	6	ENSDART00000115280
KIAA1755	ENSG00000149633	*quo*	ENSDARG00000073684			2	5	ENSDART00000109679
KIAA1755	ENSG00000149633	*si:dkey-65j6.2*	ENSDARG00000103586			3	5	ENSDART00000158832

Table showing zebrafish orthologues of human candidate genes and their main transcript. Target %id is the percentage of the orthologous sequence that matches the human sequence (Ensembl); Query %id is the percentage of the human sequence matching the sequence of the orthologue; Conservation shows the number of genes in the locus that are preserved in the same locus across species according to Genomicus.

Supplementary Table 2: Overview of CRISPR/Cas9 guide RNAs

Human gene	ENSG stable ID	Zebrafish orthologue	ENSDARG stable ID	Main transcript	Target sequence gRNA	Exon target (transcript name)	CRISPR-scan rank	ChopChop rank	Activity test rank	Predicted off-targets	Nr of mismatches	Genomic location of predicted off-target 1	ENSDARG predicted off-target 1, Feature	ENSG / gene name) predicted off-target 1	Genomic location predicted off-target 2	ENSDARG (gene name) predicted off-target 2, Feature	ENSG predicted off-target 2	Primer forward	Primer reverse	Amplicon size
GNGT1 / GNG11	ENSG00000127928 / ENSG00000127920	*gngt1*	ENSDARG00000035798	ENSDART00000051950	gGACCTGGATAAGGCGAAGA	2/3 (gngt1-001); 1/2 (gngt1-002)	62	4/13 (average)	moderate	1	3	chr25:17488676	ENSDARG00000051959; *mmp15a*; intron (3/6), intron (4/4)	no human orthologue				GTCCATTTTCCAGAGTGGAGAC	gCCCATAACACAATTGCTTACC	207
SYT10	ENSG00000110975	*syt10*	ENSDARG00000045750	ENSDART00000136049	GGCTGATGCCGTCCTCTGTG	1/7 (syt10-001); 1/7 (syt10-201)	36	6/129 (good)	low	0								TAGGATATGGACTTGGACGCAT	TTACCTGGTATGTTGCTCTCCA	227
RGS6		*rgs6*	ENSDARG00000015627	ENSDART00000135513	GGCCTCTCAGGACCAAGGTG	1/17 (rgs6-201); 2/18 (rgs6-001)	62	1/107 (good)	low	0								AAGGCTAATTCATTGTCTGGGA	GCATGTCAAATGTAAAGGCAAA	169
HCN4	ENSG00000138622	*hcn4*	ENSDARG00000061685	ENSDART00000136140	GGAGAGCCCTCCTGGAGCCG	1/8 (hcn4-001)	59	11/280 (good)	high	2	3/3	chr21:45844184	ENSDARG00000103825; *galnt10*; exon (1/3)	ENSG00000164574 (*GALNT10*)	chrUn_KN150614v1:3424	ENSDARG00000100453; (*galnt10*); exon (6/8)	ENSG00000164574 (*GALNT10*)	CCTTTCGTCCATCACCAGTC	CCGCTTGATAATACCTCTCGTC	230
HCN4	ENSG00000138622	*hcn4l*	ENSDARG00000074419	ENSDART00000088249	gGCCTGGATAGTGTTTAACG	2/8 (hcn4l-201); 1/7 (hcn4l-001)	/	2/191 (good)	low	0								TTACTGGGACCTGATCATGCTT	AAGATGTAATCCACAGGGATGG	235
NEO1	ENSG00000067141	*neo1a*	ENSDARG00000102855	ENSDART00000158160	GGAGCCGTCGGATACACTAG	1/4 (neo1a-001); 2/29 (neo1a-201); 2/30 (neo1a-202)	48	2/55 (good)	very high	0								TTCTGTGTCTCCACAGGATCAG	CCTCATCGGGTTTATTGTGTTT	215
NEO1	ENSG00000067141	*neo1b*	ENSDARG00000075100	ENSDART00000115280	GGACAGAGATGCTCGGCCTG	3/29 (neo1b-201)	58	18/354 (good)	very high	0								CAGCTCTCTCTCTCATTGGCTT	TATGGTGCAACGGTAGAGTCC	206
KIAA1755	ENSG00000149633	*quo*	ENSDARG00000073684	ENSDART00000109679	GGAGATCCGGTTGCCCTGTG	2/22 (quo-001); 3/23 (quo-201)	83	12/331 (good)	very high	0								ATGACCGTAAGTCAAGGGAAAA	TGAATCTTGTCTAATGGCGTTG	153
KIAA1755	ENSG00000149633	*si:dkey-65j6.2*	ENSDARG00000103586	ENSDART00000158832	GGACGGTTTGGCTCCAGCAG	1/23 (si:dkey-65j6.2-201); 3/25 (si:dkey-65j6.2-001)	59	14/303 (good)	very high	2	3/3	chr15:32969618	ENSDARG00000104664; *dclk1b*; exon (3/16), exon (4/17)	ENSG00000133083 (*DCLK1*)	chr22:28819880	ENSDARG00000071083; (*si:dkeyp-34c12.1*); Intron (7/7), Exon (8/9)	no human orthologue	AGCCATTACACCCAAAGAAAAA	GGGAATCATTTCGAAGTAGCTG	152

Table showing information on the CRISPR/Cas9 gRNAs used to target the zebrafish orthologues of human candidate genes. A lower case "g" in the gRNA target sequence indicates a manually introduced nucleotide change to guanine; Exon target shows which exon was targeted for each of the orthologues' transcripts; ChopChop rank indicates the rank of the gRNA as per the online tool ChopChop and its rating (good or average); Activity test rank quantification shows results from a fragment length PCR analysis in eight injected embryos at 3 days post-fertilization: Low: 8 of 8 larvae showed wildtype sequence and fewer than 4 of 8 also showed an indel sequence, Moderate: 8 of 8 larvae showed the wildtype sequence and >4 of 8 also showed an indel sequence, High: 8 of 8 larvae showed wildtype as well as indel sequences, Very high: Fewer than 4 of 8 larvae showed wildtype sequence and all larvae showed indel sequences; ENSDARG of potential off-target refers to the Ensembl gene ID of the potential off-target and where the potential target is located (Feature), *mmp15a* and *si:dkeyp-34c12.1* have no human orthologue, whereas *galnt10* and *dckl1b* may exert an effect on heart rate and/or rhythm. However, no CRISPR/Cas9-induced mutations were identified at the predicted off-target sites in the F_1 embryos included in the screen.

Supplementary Table 3: Unique variants and affected transcripts for each targeted zebrafish orthologue

Orthologue	Chr	ENSDARG	Affected transcripts	Start (bp)	End (bp)	Mutation	Annotation	Base pair change	VEP impact	Nr affected alleles
gngt1	19	ENSDARG00000035798	ENSDART00000051950<br>ENSDART00000139083	40,856,888	40,856,893	AAGGCG/-	inframe deletion	-6	moderate	75
				40,856,893	40,856,895	GAA/-	inframe deletion	-3	moderate	3
				40,856,894	40,856,894	A/-	frameshift variant	-1	high	69
				40,856,895	40,856,895	A/T	missense variant	0	moderate	3
				40,856,895	40,856,897	AGA/TTT	missense variant	0	moderate	69
				40,856,897	40,856,896	-/TAAC	stop gained,frameshift variant	4	high	2
				40,856,897	40,856,899	ATG/CCA	missense variant	0	moderate	2
syt10	4	ENSDARG00000045750	ENSDART00000136049	10,888,933	10,888,953	GACATGAATGTCCGCACAGAG/-	inframe deletion	-21	moderate	19
				10,888,944	10,888,944	C/A	synonymous variant	0	low	42
				10,888,945	10,888,944	-/CAGCTGATG	inframe insertion	9	moderate	42
				10,888,946	10,888,950	GCACA/-	frameshift variant	-5	high	3
				10,888,946	10,888,952	GCACAGA/-	frameshift variant	-7	high	15
				10,888,946	10,888,953	GCACAGAG/-	frameshift variant	-8	high	40
				10,888,947	10,888,947	C/T	synonymous variant	0	low	42
				10,888,948	10,888,948	A/C	missense variant	0	moderate	4
				10,888,948	10,888,954	ACAGAGG/-	frameshift variant	-7	high	11
				10,888,949	10,888,958	CAGAGGACGG/-	frameshift variant	-10	high	4
				10,888,950	10,888,949	-/GGGAATGG	frameshift variant	8	high	42
				10,888,950	10,888,949	-/A	frameshift variant	1	high	44
				10,888,950	10,888,950	A/G	synonymous variant	0	low	11
				10,888,951	10,888,951	G/T	stop gained	0	high	42
				10,888,952	10,888,951	-/CAT	stop gained,protein altering variant	3	high	11
				10,888,952	10,888,952	A/-	frameshift variant	-1	high	42
				10,888,952	10,888,953	AG/CA	missense variant	0	moderate	11
				10,888,959	10,888,960	CA/TG	missense variant	0	moderate	4
rgs6	20	ENSDARG00000015627	ENSDART00000135513<br>ENSDART00000184779	28,638,811	28,638,822	CCCACCGCACCT/-	inframe deletion	-12	moderate	1
				28,638,820	28,638,820	C/-	frameshift variant	-1	high	24
				28,638,820	28,638,821	CC/-	frameshift variant	-2	high	7
				28,638,820	28,638,822	CCT/-	inframe deletion	-3	moderate	4
				28,638,824	28,638,824	G/A	stop gained	0	high	4
				28,638,837	28,638,836	-/CTGAGCCATG	frameshift variant	10	high	4
hcn4	18	ENSDARG00000061685	ENSDART00000136140<br>ENSDART00000189186	1,143,637	1,143,644	ACCCGCGG/-	frameshift variant	-8	high	13
				1,143,638	1,143,638	C/T	missense variant	0	moderate	16
				1,143,641	1,143,641	G/A	missense variant	0	moderate	9
				1,143,643	1,143,643	G/-	frameshift variant	-1	high	11
				1,143,643	1,143,644	GG/CA	missense variant	0	moderate	2
				1,143,643	1,143,646	GGCT/-	frameshift variant	-4	high	9
				1,143,644	1,143,643	-/CCGCCAC	frameshift variant	7	high	16
				1,143,644	1,143,644	G/C	missense variant	0	moderate	75
				1,143,644	1,143,646	GCT/-	inframe deletion	-3	moderate	1
				1,143,645	1,143,644	-/CTCTC	frameshift variant	5	high	15
				1,143,645	1,143,644	-/GCC	protein altering variant	3	moderate	2
				1,143,645	1,143,645	C/-	frameshift variant	-1	high	2
				1,143,646	1,143,645	-/GCCA	frameshift variant	4	high	48
				1,143,646	1,143,646	T/C	synonymous variant	0	low	48
				1,143,652	1,143,651	-/AGCTCC	inframe insertion	6	moderate	15

Continued Supplementary Table 3

Orthologue	Chr	ENSDARG	Affected transcripts	Start (bp)	End (bp)	Mutation	Annotation	Base pair change	VEP impact	Nr affected alleles
hcn4l	25	ENSDARG00000074419	ENSDART00000088249 ENSDART00000148940	29,273,086	29,273,101	CCACGTTAAACACTAT/-	frameshift variant	-16	high	9
				29,273,091	29,273,090	-/T	frameshift variant	1	high	4
				29,273,092	29,273,093	TA/-	frameshift variant	-2	high	18
neo1a	7	ENSDARG00000102855	ENSDART00000158160 ENSDART00000163261 ENSDART00000164768 ENSDART00000181629	53,879,228	53,879,244	CACGGAGCCGTCGGATA/-	frameshift variant	-17	high	1
				53,879,231	53,879,258	GGAGCCGTCGGATACACTAGCGGTGCGA/-	frameshift variant	-28	high	11
				53,879,235	53,879,265	CCGTCGGATACACTAGCGGTGCGAGGAGCGC/-	frameshift variant	-31	high	57
				53,879,237	53,879,252	GTCGGATACACTAGCG/-	frameshift variant	-16	high	157
				53,879,237	53,879,267	GTCGGATACACTAGCGGTGCGAGGAGCGCCA/-	frameshift variant	-31	high	9
				53,879,239	53,879,250	CGGATACACTAG/-	inframe deletion	-12	moderate	66
				53,879,240	53,879,246	GGATACA/-	frameshift variant	-7	high	2
				53,879,242	53,879,248	ATACACT/-	frameshift variant	-7	high	5
				53,879,243	53,879,247	TACAC/-	frameshift variant	-5	high	17
				53,879,243	53,879,268	TACACTAGCGGTGCGAGGAGCGCCAG/-	frameshift variant	-26	high	16
				53,879,244	53,879,245	AC/GT	missense variant	0	moderate	1
				53,879,245	53,879,246	CA/GT	missense variant	0	moderate	5
				53,879,245	53,879,255	CACTAGCGGTG/-	frameshift variant	-11	high	20
				53,879,246	53,879,248	ACT/-	inframe deletion	-3	moderate	20
				53,879,247	53,879,246	-/GCGGTGCGAGGAGT	frameshift variant	14	high	1
				53,879,247	53,879,246	-/TCCAT	frameshift variant	5	high	14
				53,879,247	53,879,247	C/G	missense variant	0	moderate	1
				53,879,247	53,879,248	CT/TC	missense variant	0	moderate	14
				53,879,247	53,879,250	CTAG/-	frameshift variant	-4	high	82
				53,879,247	53,879,252	CTAGCG/-	inframe deletion	-6	moderate	10
				53,879,247	53,879,255	CTAGCGGTG/-	inframe deletion	-9	moderate	69
				53,879,248	53,879,247	-/GGTGCG	protein altering variant	6	moderate	6
				53,879,248	53,879,248	T/G	missense variant	0	moderate	6
				53,879,248	53,879,248	T/C	missense variant	0	moderate	10
				53,879,248	53,879,258	TAGCGGTGCGA/-	frameshift variant	-11	high	5
				53,879,249	53,879,249	A/G	synonymous variant	0	low	2
				53,879,249	53,879,260	AGCGGTGCGAGG/-	inframe deletion	-12	moderate	5
				53,879,250	53,879,254	GCGGT/-	frameshift variant	-5	high	2
				53,879,253	53,879,254	GT/AA	missense variant	0	moderate	10
				53,879,262	53,879,265	GCGC/-	frameshift variant	-4	high	5
				53,879,263	53,879,262	-/CGCCAGTTCT	frameshift variant	10	high	89
				53,879,266	53,879,266	C/T	missense variant	0	moderate	5

Continued Supplementary Table 3

Orthologue	Chr	ENSDARG	Affected transcripts	Start (bp)	End (bp)	Mutation	Annotation	Base pair change	VEP impact	Nr affected alleles
neo1b	25	ENSDARG00000075100	ENSDART00000115280	2,899,719	2,899,719	C/-	frameshift variant	-1	high	65
				2,899,722	2,899,721	-/GGTC	frameshift variant	4	high	18
				2,899,719	2,899,718	-/TCGG	frameshift variant	4	high	51
				2,899,710	2,899,728	ATGCTCGGCCTGTGGAGCT/-	frameshift variant	-19	high	7
				2,899,718	2,899,724	CCTGTGG/-	frameshift variant	-7	high	9
				2,899,716	2,899,719	GGCC/-	frameshift variant	-4	high	15
				2,899,708	2,899,729	AGATGCTCGGCCTGTGGAGCTG/-	frameshift variant	-22	high	1
				2,899,715	2,899,728	CGGCCTGTGGAGCT/-	frameshift variant	-14	high	2
				2,899,712	2,899,718	GCTCGGC/-	frameshift variant	-7	high	1
				2,899,719	2,899,718	-/AGCAGGGT	frameshift variant	8	high	18
				2,899,719	2,899,718	-/AG	frameshift variant	2	high	1
				2,899,716	2,899,732	GGCCTGTGGAGCTGGAC/-	frameshift variant	-17	high	5
				2,899,719	2,899,724	CTGTGG/-	inframe deletion	-6	moderate	59
				2,899,713	2,899,721	CTCGGCCTG/-	inframe deletion	-9	moderate	18
				2,899,722	2,899,723	TG/AT	missense variant	0	moderate	2
				2,899,725	2,899,726	AG/CT	missense variant	0	moderate	2
				2,899,734	2,899,737	GCAG/TTCA	missense variant	0	moderate	7
				2,899,728	2,899,729	TG/AT	missense variant	0	moderate	2
				2,899,716	2,899,718	GGC/ATT	missense variant	0	moderate	2
				2,899,715	2,899,720	CGGCCT/-	inframe deletion	-6	moderate	108
				2,899,740	2,899,740	T/A	missense variant	0	moderate	7
				2,899,720	2,899,720	T/A	synonymous variant	0	low	2

Continued Supplementary Table 3

Orthologue	Chr	ENSDARG	Affected transcripts	Start (bp)	End (bp)	Mutation	Annotation	Base pair change	VEP impact	Nr affected alleles
				2,051,654	2,051,734	ATGACCCAACTTATCACAAGCATGGTCCAAGCAAGACAGCATCAC AAGTCATTCCTCACAGGGCAACCGGATCTCCCTCAG/-	inframe deletion	-81	moderate	13
				2,051,699	2,051,699	A/T	stop gained	0	high	10
				2,051,701	2,051,715	GTCATTCCTCACAGG/-	inframe deletion	-15	moderate	10
				2,051,703	2,051,716	CATTCCTCACAGGG/-	frameshift variant	-14	high	61
				2,051,706	2,051,708	TCC/-	inframe deletion	-3	moderate	17
				2,051,709	2,051,715	TCACAGG/-	frameshift variant	-7	high	9
				2,051,710	2,051,716	CACAGGG/-	frameshift variant	-7	high	53
				2,051,710	2,051,719	CACAGGGCAA/-	frameshift variant	-10	high	51
				2,051,712	2,051,711	-/CCGG	frameshift variant	4	high	1
				2,051,712	2,051,711	-/ACCGGACT	frameshift variant	8	high	7
				2,051,712	2,051,712	C/T	missense variant	0	moderate	15
				2,051,712	2,051,714	CAG/ACC	missense variant	0	moderate	9
				2,051,712	2,051,716	CAGGG/-	frameshift variant	-5	high	14
			ENSDART00000109679	2,051,712	2,051,728	CAGGGCAACCGGATCTC/-	frameshift variant	-17	high	23
			ENSDART00000153568	2,051,713	2,051,712	-/G	frameshift variant	1	high	15
quo	6	ENSDARG00000073684	ENSDART00000187502	2,051,713	2,051,712	-/A	frameshift variant	1	high	19
			ENSDART00000187544	2,051,713	2,051,712	-/TTG	stop gained,inframe insertion	3	high	70
			ENSDART00000191165	2,051,713	2,051,713	A/C	synonymous variant	0	low	22
				2,051,713	2,051,714	AG/TA	missense variant	0	moderate	70
				2,051,713	2,051,716	AGGG/-	frameshift variant	-4	high	4
				2,051,714	2,051,713	-/ACC	inframe insertion	3	moderate	51
				2,051,714	2,051,714	G/-	frameshift variant	-1	high	22
				2,051,714	2,051,714	G/A	missense variant	0	moderate	40
				2,051,714	2,051,714	G/C	missense variant	0	moderate	15
				2,051,715	2,051,714	-/C	frameshift variant	1	high	22
				2,051,715	2,051,715	G/C	missense variant	0	moderate	22
				2,051,716	2,051,716	G/T	synonymous variant	0	low	18
				2,051,716	2,051,716	G/A	synonymous variant	0	low	10
				2,051,719	2,051,720	AC/TT	missense variant	0	moderate	1
				2,051,719	2,051,723	ACCGG/-	frameshift variant	-5	high	17
				2,051,724	2,051,724	A/T	missense variant	0	moderate	17
				2,051,736	2,051,736	C/A	missense variant	0	moderate	13

Continued Supplementary Table 3

Orthologue	Chr	ENSDARG	Affected transcripts	Start (bp)	End (bp)	Mutation	Annotation	Base pair change	VEP impact	Nr affected alleles
si:dkey-65j6.	23	ENSDARG00000103586	ENSDART00000158832<br>ENSDART00000164262<br>ENSDART00000186065<br>ENSDART00000190315<br>ENSDART00000193300	43,213,133	43,213,153	GGACGGTTTGGCTCCAGCAGT/-	inframe deletion	-21	moderate	62
				43,213,135	43,213,160	ACGGTTTGGCTCCAGCAGTGGTCGGC/-	frameshift variant	-26	high	1
				43,213,136	43,213,149	CGGTTTGGCTCCAG/-	frameshift variant	-14	high	21
				43,213,136	43,213,156	CGGTTTGGCTCCAGCAGTGGT/-	inframe deletion	-21	moderate	101
				43,213,136	43,213,164	CGGTTTGGCTCCAGCAGTGGTCGGCACAT/-	frameshift variant	-29	high	50
				43,213,137	43,213,153	GGTTTGGCTCCAGCAGT/-	frameshift variant	-17	high	15
				43,213,138	43,213,138	G/A	missense variant	0	moderate	4
				43,213,138	43,213,168	GTTTGGCTCCAGCAGTGGTCGGCACATCAGA/-	frameshift variant	-31	high	3
				43,213,139	43,213,139	T/G	synonymous variant	0	low	18
				43,213,139	43,213,143	TTTGG/CACAT	missense variant	0	moderate	9
				43,213,140	43,213,140	T/A	missense variant	0	moderate	3
				43,213,140	43,213,147	TTGGCTCC/-	frameshift variant	-8	high	18
				43,213,140	43,213,151	TTGGCTCCAGCA/-	inframe deletion	-12	moderate	7
				43,213,140	43,213,160	TTGGCTCCAGCAGTGGTCGGC/-	inframe deletion	-21	moderate	12
				43,213,141	43,213,152	TGGCTCCAGCAG/-	inframe deletion	-12	moderate	32
				43,213,142	43,213,141	-/AAAAATAA	frameshift variant	8	high	7
				43,213,142	43,213,157	GGCTCCAGCAGTGGTC/-	frameshift variant	-16	high	3
				43,213,143	43,213,153	GCTCCAGCAGT/-	frameshift variant	-11	high	13
				43,213,143	43,213,157	GCTCCAGCAGTGGTC/-	inframe deletion	-15	moderate	57
				43,213,144	43,213,144	C/-	frameshift variant	-1	high	1
				43,213,145	43,213,149	TCCAG/-	frameshift variant	-5	high	9
				43,213,146	43,213,146	C/-	frameshift variant	-1	high	41
				43,213,146	43,213,150	CCAGC/-	frameshift variant	-5	high	7
				43,213,146	43,213,161	CCAGCAGTGGTCGGCA/-	frameshift variant	-16	high	34
				43,213,147	43,213,147	C/G	missense variant	0	moderate	1
				43,213,147	43,213,149	CAG/-	inframe deletion	-3	moderate	69
				43,213,149	43,213,149	G/T	missense variant	0	moderate	41
				43,213,149	43,213,149	G/C	missense variant	0	moderate	18
				43,213,149	43,213,149	G/A	missense variant	0	moderate	1
				43,213,152	43,213,152	G/A	missense variant	0	moderate	3
				43,213,153	43,213,153	T/A	missense variant	0	moderate	41
				43,213,155	43,213,154	-/AAACACAGAGA	frameshift variant	11	high	41
				43,213,155	43,213,157	GTC/AAT	missense variant	0	moderate	41
				43,213,159	43,213,160	GC/-	frameshift variant	-2	high	3

The impact of each CRISPR/Cas9-induced mutation on protein function was predicted using Ensembl's Variant Effect Predictor (VEP). An allele- and target-specific dosage score was subsequently calculated for each embryo by weighting the mutation with the highest predicted impact on protein function by a factor 0.33, 0.66, or 1 for mutations with a low, moderate or high predicted impact, respectively, followed by summing the score across both alleles for each targeted site in each embryo. Start and end coordinates are based on GRCz11. The genes *quo* and *si:dkey-65j6.2* are orthologues of the human *KIAA1755* .

Supplementary Table 4: Mutant allele frequencies for CRISPR/Cas9-induced mutations by zebrafish orthologue

	Nr mutated alleles							Expected nr mutated alleles			
**Gene**	**0**	**1**	**2**	**2***	**Missing**	**Sample call rate**	**Mutant allele freq**	**0**	**1**	**2**	P_{HWE}
gngt1	234	130	12	2	5	0.987	0.205	238	122	16	2.33E-01
syt10	192	130	54	46	5	0.987	0.316	176	163	38	9.82E-05
rgs6	344	34	1	1	2	0.995	0.047	344	34	1	8.69E-01
hcn4	266	100	9	9	6	0.984	0.157	266	99	9	9.12E-01
hcn4l	349	29	1	1	2	0.995	0.041	349	30	1	6.32E-01
neo1a	19	113	240	109	9	0.976	0.797	15	120	236	2.39E-01
neo1b	14	12	277	116	78	0.795	0.934	1	37	264	3.24E-32
quo	55	42	281	221	3	0.992	0.799	15	121	241	4.69E-37
si:dkey-65j6.2	22	60	295	66	4	0.990	0.862	7	90	280	1.34E-10

Number of embryos with 0, 1 and 2 mutated alleles; the number of embryos with nonsense mutations in both alleles (2*); and the number of embryos with a missing call for that gene. P_{HWE}: P-value for a Hardy-Weinberg equilibrium (HWE) exact test, considering a ±30 bp window around the CRISPR/Cas9-targeted site as a single locus ($P<2.9x10^{-3}$ is significant after Bonferroni correction). In cases of deviation from HWE, the number of embryos with 2 mutated alleles is not lower than expected. Embryos with missing sequencing calls at more than two targeted orthologues were excluded from the statistical analysis (i.e. two embryos). For all genes, missed calls were normally distributed throughout the HRV and heart rate distributions, so for embryos with missed calls in at most two genes, we imputed the mean call for that gene, including for *neo1b* , where exclusion of either the gene or the embryos with a missed call was

Supplementary Table 5: Additive effects of CRISPR/Cas9-induced mutations on sinoatrial pauses and arrests

Age	Outcome	n controls	n cases	Gene	OR	LCI	UCI	P
2dpf	SA pause	258	39	*gngt1*	1.163	0.494	2.740	7.29E-01
				syt10	1.467	0.810	2.654	2.06E-01
				rgs6	0.788	0.175	3.553	7.57E-01
				hcn4	2.749	1.461	5.171	1.71E-03
				hcn4l	omittted			
				neo1a	1.176	0.507	2.731	7.06E-01
				neo1b	2.445	0.658	9.080	1.82E-01
				quo	0.746	0.401	1.389	3.56E-01
				si:dkey-65j6.2	0.755	0.339	1.685	4.93E-01
				time of day	1.147	0.856	1.539	3.59E-01
				intercept	0.004	0.000	0.185	4.77E-03
				Batch 1	7.380	0.755	72.106	8.57E-02
				Batch 2	14.723	1.714	126.443	1.42E-02
				Batch 3	5.507	0.633	47.917	1.22E-01
				Batch 4	4.948	0.457	53.573	1.88E-01
				Batch 5	0.890	0.042	18.778	9.40E-01
2dpf	SA arrest	212	36	*gngt1*	0.951	0.387	2.336	9.12E-01
				syt10	1.447	0.792	2.642	2.30E-01
				rgs6	0.855	0.193	3.799	8.37E-01
				hcn4	2.644	1.402	4.986	2.66E-03
				hcn4l	omitted			
				neo1a	1.165	0.499	2.717	7.24E-01
				neo1b	1.694	0.434	6.604	4.48E-01
				quo	0.764	0.404	1.442	4.06E-01
				si:dkey-65j6.2	0.750	0.327	1.720	4.97E-01
				time of day	1.117	0.829	1.506	4.66E-01
				intercept	0.008	0.000	0.414	1.64E-02
				Batch 1	7.540	0.770	73.835	8.27E-02
				Batch 2	11.656	1.342	101.224	2.60E-02
				Batch 3	5.798	0.665	50.571	1.12E-01
				Batch 4	5.656	0.523	61.178	1.54E-01
				Batch 5	omitted			
5dpf	SA pause	312	9	*gngt1*	0.309	0.037	2.572	2.77E-01
				syt10	0.236	0.050	1.128	7.05E-02
				rgs6	1.049	0.068	16.223	9.73E-01
				hcn4	2.460	0.680	8.899	1.70E-01
				hcn4l	0.937	0.086	10.181	9.58E-01
				neo1a	0.897	0.190	4.232	8.91E-01
				neo1b	1.561	0.162	15.023	7.00E-01
				quo	0.672	0.221	2.043	4.84E-01
				si:dkey-65j6.2	3.533	0.421	29.662	2.45E-01
				time of day	1.066	0.514	2.209	8.64E-01
				intercept	0.016	0.000	7.788	1.90E-01
				Batch 1	0.240	0.011	5.447	3.70E-01
				Batch 2	0.703	0.073	6.750	7.60E-01
				Batch 3	0.214	0.015	3.049	2.55E-01
				Batch 5	0.213	0.005	8.956	4.18E-01

Associations of dichotomous cardiac outcomes with the number of mutated alleles across each of the nine orthologues, weighted by their predicted effect on protein function. At 2 and 5 days post fertilization (dpf), associations were analyzed using logistic regression for outcomes with at least 10 cases. Associations were adjusted for time of day and for the weighted number of mutated alleles in the other genes as fixed factors. The genes *quo* and *si:dkey-65j6.2* are orthologues of the human *KIAA1755* .

Supplementary Table 6: Effects of CRISPR/Cas9-induced nonsense mutations in both alleles vs. no CRISPR/Cas9-induced mutations on sinoatrial pauses and arrests at 2dpf

Gene	Outcome	n controls	n cases	OR	LCI	UCI	P
syt10	SA pause	162	21	3.451	0.792	15.043	9.92E-02
	SA arrest	121	20	3.480	0.789	15.347	9.95E-02
hcn4	SA pause	127	23	3.667	0.633	21.249	1.47E-01
	SA arrest	128	21	3.666	0.655	20.525	1.39E-01
neo1a	SA pause	56	16	1.200	0.148	9.717	8.65E-01
	SA arrest	56	16	1.200	0.148	9.717	8.65E-01
quo	SA pause	158	32	0.513	0.132	1.993	3.35E-01
	SA arrest	121	29	0.553	0.136	2.239	4.06E-01
si:dkey-65j6.2	SA pause	39	8	0.145	0.009	2.389	1.77E-01
	SA arrest	39	8	0.145	0.009	2.389	1.77E-01

Associations were examined using logistic regression analyses for outcomes with at least 10 cases and for genes with at least 5 embryos with a nonsense mutation in both alleles. Associations were adjusted for time of day, batch, and the weighted number of mutated alleles in the other genes. Models could not be adjusted for the weighted number of mutated alleles in *hcn4l* , since none of the embryos with a mutated *hcn4l* allele showed a sinoatrial pause or arrest. Furthermore, data from the first and/or last round of the experiment were typically excluded from the analysis because no embryos showed a sinoatrial pause or arrest. The genes *quo* and *si:dkey-65j6.2* are orthologues of the human *KIAA1755* .

Supplementary Table 7: Additive effects of CRISPR/Cas9-induced mutations on (change in) heart rate variability and heart rate

Age	n	Outcome	Factor	Gene	Model 1					Model 2				
					Effect size	SE	LCI	UCI	P	Effect size	SE	LCI	UCI	P
2dpf	234	HRV	fixed	*gngt1*	0.155	0.141	-0.120	0.431	2.69E-01	0.112	0.124	-0.131	0.354	3.66E-01
				syt10	-0.027	0.096	-0.214	0.161	7.78E-01	-0.032	0.084	-0.197	0.132	7.00E-01
				rgs6	0.270	0.230	-0.181	0.720	2.41E-01	0.031	0.204	-0.370	0.431	8.80E-01
				hcn4	0.246	0.129	-0.008	0.499	5.75E-02	0.209	0.114	-0.015	0.432	6.71E-02
				hcn4l	0.083	0.200	-0.309	0.476	6.78E-01	0.110	0.176	-0.236	0.455	5.34E-01
				neo1a	0.011	0.139	-0.261	0.283	9.37E-01	0.046	0.123	-0.195	0.288	7.06E-01
				neo1b	0.172	0.133	-0.088	0.432	1.94E-01	0.172	0.117	-0.057	0.400	1.41E-01
				quo	0.116	0.097	-0.074	0.307	2.32E-01	0.072	0.086	-0.097	0.241	4.03E-01
				si:dkey-65j6.2	0.188	0.121	-0.050	0.426	1.21E-01	0.221	0.107	0.012	0.431	3.87E-02
				time of day at 2dpf	0.296	0.047	0.204	0.388	2.80E-10	0.526	0.050	0.428	0.624	5.05E-26
				Heart rate	-	-	-	-	-	-0.513	0.062	-0.636	-0.391	2.07E-16
				intercept	-1.718	0.381	-2.466	-0.970	6.68E-06	-2.362	0.353	-3.054	-1.670	2.20E-11
			random	variation by batch	0.310	0.128	0.138	0.697	-	0.306	0.119	0.143	0.657	-
				residual	0.869	0.041	0.792	0.952	-	0.763	0.036	0.696	0.837	-
		heart rate	fixed	*gngt1*	-0.080	0.131	-0.337	0.176	5.41E-01	-0.009	0.116	-0.236	0.217	9.37E-01
				syt10	-0.003	0.088	-0.176	0.170	9.75E-01	-0.006	0.078	-0.159	0.147	9.38E-01
				rgs6	-0.477	0.213	-0.895	-0.060	2.49E-02	-0.376	0.188	-0.745	-0.008	4.53E-02
				hcn4	-0.043	0.116	-0.271	0.184	7.08E-01	0.023	0.105	-0.182	0.227	8.29E-01
				hcn4l	0.096	0.182	-0.259	0.452	5.96E-01	0.148	0.162	-0.169	0.465	3.59E-01
				neo1a	0.110	0.118	-0.121	0.341	3.49E-01	0.138	0.107	-0.072	0.348	1.99E-01
				neo1b	0.006	0.120	-0.230	0.241	9.61E-01	0.062	0.107	-0.149	0.272	5.65E-01
				quo	-0.076	0.084	-0.240	0.088	3.64E-01	0.000	0.076	-0.149	0.149	1.00E+00
				si:dkey-65j6.2	0.059	0.108	-0.152	0.270	5.84E-01	0.167	0.097	-0.023	0.358	8.49E-02
				time of day at 2dpf	0.459	0.042	0.378	0.541	2.07E-28	0.579	0.040	0.500	0.658	1.55E-46
				HRV	-	-	-	-	-	-0.424	0.052	-0.526	-0.321	6.06E-16
				intercept	-1.353	0.280	-1.902	-0.805	1.33E-06	-2.148	0.285	-2.706	-1.590	4.47E-14
			random	variation by batch	0.031	0.233	0.000	66000	-	0.098	0.097	0.014	0.680	-
				residual	0.810	0.038	0.738	0.889	-	0.712	0.034	0.649	0.781	-
5dpf	285	HRV	fixed	*gngt1*	0.125	0.133	-0.136	0.386	3.47E-01	0.130	0.109	-0.084	0.344	2.35E-01
				syt10	-0.063	0.089	-0.237	0.111	4.76E-01	-0.066	0.074	-0.211	0.078	3.69E-01
				rgs6	0.231	0.192	-0.145	0.608	2.29E-01	0.100	0.159	-0.212	0.412	5.30E-01
				hcn4	-0.286	0.112	-0.506	-0.066	1.09E-02	-0.129	0.096	-0.317	0.059	1.78E-01
				hcn4l	0.056	0.211	-0.358	0.470	7.90E-01	0.080	0.179	-0.272	0.431	6.56E-01
				neo1a	0.117	0.118	-0.114	0.348	3.22E-01	0.035	0.103	-0.167	0.237	7.34E-01
				neo1b	-0.014	0.139	-0.286	0.257	9.17E-01	0.002	0.116	-0.226	0.231	9.83E-01
				quo	0.072	0.092	-0.107	0.251	4.31E-01	0.053	0.080	-0.103	0.209	5.06E-01
				si:dkey-65j6.2	0.243	0.119	0.010	0.476	4.08E-02	0.173	0.101	-0.024	0.370	8.59E-02
				time of day at 5dpf	0.241	0.050	0.143	0.339	1.51E-06	0.521	0.050	0.423	0.620	2.36E-25
				Heart rate	-	-	-	-	-	-0.601	0.053	-0.705	-0.498	3.75E-30
				intercept	-1.293	0.366	-2.010	-0.575	4.14E-04	-1.981	0.386	-2.738	-1.223	2.97E-07
			random	variation by batch	0.145	0.092	0.042	0.500	-	0.477	0.170	0.237	0.959	-
				residual	0.915	0.039	0.842	0.994	-	0.751	0.032	0.692	0.816	-
		heart rate	fixed	*gngt1*	0.001	0.123	-0.240	0.242	9.96E-01	0.068	0.101	-0.130	0.266	5.01E-01
				syt10	-0.018	0.083	-0.181	0.145	8.31E-01	-0.049	0.068	-0.183	0.085	4.75E-01
				rgs6	-0.170	0.179	-0.520	0.181	3.43E-01	-0.062	0.147	-0.351	0.227	6.75E-01
				hcn4	0.338	0.106	0.130	0.545	1.44E-03	0.157	0.089	-0.016	0.331	7.58E-02
				hcn4l	-0.035	0.202	-0.430	0.360	8.63E-01	0.024	0.166	-0.301	0.350	8.83E-01
				neo1a	-0.139	0.115	-0.365	0.087	2.27E-01	-0.090	0.095	-0.277	0.097	3.47E-01
				neo1b	0.136	0.131	-0.121	0.392	3.00E-01	0.083	0.108	-0.128	0.294	4.42E-01
				quo	0.011	0.089	-0.165	0.186	9.04E-01	0.022	0.074	-0.123	0.168	7.62E-01
				si:dkey-65j6.2	-0.076	0.113	-0.297	0.146	5.02E-01	0.030	0.094	-0.153	0.214	7.48E-01
				time of day at 5dpf	0.410	0.051	0.310	0.509	6.80E-16	0.565	0.044	0.479	0.651	5.89E-38
				HRV	-	-	-	-	-	-0.524	0.045	-0.613	-0.435	7.90E-31
				intercept	-1.243	0.421	-2.069	-0.417	3.17E-03	-1.858	0.406	-2.654	-1.063	4.70E-06
			random	variation by batch	0.509	0.181	0.253	1.023	-	0.615	0.206	0.319	1.186	-
				residual	0.845	0.036	0.778	0.918	-	0.694	0.029	0.639	0.754	-
Δ5dpf - 2dpf	197	ΔHRV	fixed	*gngt1*	0.004	0.156	-0.301	0.310	9.78E-01	-0.025	0.131	-0.282	0.231	8.46E-01
				syt10	0.003	0.105	-0.202	0.208	9.79E-01	-0.033	0.088	-0.205	0.140	7.11E-01
				rgs6	-0.049	0.245	-0.530	0.431	8.40E-01	-0.054	0.206	-0.458	0.349	7.92E-01
				hcn4	-0.482	0.137	-0.751	-0.213	4.43E-04	-0.317	0.117	-0.547	-0.087	6.99E-03
				hcn4l	0.091	0.230	-0.360	0.543	6.92E-01	-0.025	0.194	-0.406	0.356	8.98E-01
				neo1a	0.130	0.148	-0.159	0.420	3.77E-01	0.124	0.125	-0.121	0.369	3.22E-01
				neo1b	-0.224	0.152	-0.523	0.075	1.42E-01	-0.135	0.128	-0.387	0.117	2.93E-01
				quo	-0.054	0.108	-0.265	0.158	6.19E-01	0.069	0.092	-0.111	0.250	4.53E-01
				si:dkey-65j6.2	-0.131	0.141	-0.407	0.146	3.54E-01	-0.253	0.120	-0.488	-0.018	3.47E-02
				time of day at 2dpf	-0.342	0.246	-0.824	0.140	1.65E-01	-0.286	0.252	-0.780	0.208	2.56E-01
				time of day at 5dpf	0.361	0.294	-0.216	0.937	2.20E-01	0.331	0.305	-0.268	0.929	2.79E-01
				Δheart rate	-	-	-	-	-	-0.513	0.058	-0.626	-0.399	7.54E-19
				intercept	0.335	0.559	-0.761	1.430	5.49E-01	0.140	0.527	-0.894	1.173	7.91E-01
			random	variation by batch	0.464	0.225	0.179	1.202	-	0.569	0.227	0.260	1.242	-
				residual	0.859	0.044	0.776	0.950	-	0.720	0.037	0.651	0.797	-
		Δheart rate	fixed	*gngt1*	-0.058	0.163	-0.378	0.261	7.20E-01	-0.056	0.137	-0.325	0.213	6.83E-01
				syt10	-0.076	0.109	-0.290	0.138	4.85E-01	-0.073	0.092	-0.254	0.107	4.25E-01
				rgs6	-0.036	0.256	-0.536	0.465	8.90E-01	-0.058	0.215	-0.480	0.364	7.87E-01
				hcn4	0.363	0.143	0.084	0.643	1.09E-02	0.089	0.125	-0.155	0.333	4.75E-01
				hcn4l	-0.282	0.239	-0.751	0.187	2.39E-01	-0.213	0.203	-0.610	0.184	2.94E-01
				neo1a	-0.018	0.152	-0.316	0.281	9.07E-01	0.046	0.130	-0.210	0.302	7.25E-01
				neo1b	0.171	0.159	-0.140	0.483	2.81E-01	0.045	0.135	-0.219	0.309	7.40E-01
				quo	0.235	0.111	0.018	0.453	3.41E-02	0.203	0.095	0.017	0.389	3.24E-02
				si:dkey-65j6.2	-0.269	0.146	-0.556	0.018	6.60E-02	-0.342	0.124	-0.586	-0.099	5.91E-03
				time of day at 2dpf	0.292	0.208	-0.116	0.699	1.60E-01	0.195	0.231	-0.259	0.648	4.00E-01
				time of day at 5dpf	-0.302	0.243	-0.779	0.175	2.14E-01	-0.206	0.278	-0.751	0.339	4.59E-01
				ΔHRV	-	-	-	-	-	-0.552	0.063	-0.675	-0.429	1.45E-18
				intercept	-0.094	0.533	-1.138	0.951	8.61E-01	0.167	0.508	-0.828	1.162	7.42E-01
			random	variation by batch	0.327	0.135	0.145	0.735	-	0.458	0.158	0.233	0.899	-
				residual	0.898	0.046	0.812	0.992	-	0.754	0.039	0.683	0.834	-

(Change in) heart rate variability (HRV) and heart rate were inverse normally transformed before the analysis, so effect sizes and SEs can be interpreted as z-score units. Associations of outcomes with the weighted number of mutated alleles in the main transcript of each targeted zebrafish orthologue were examined using hierarchical linear models (xtmixed in Stata). Associations were adjusted for time of day and for the weighted number of mutated alleles in the other genes as fixed factors, with embryos nested in batches (random factor). In Model 2, associations were additionally adjusted for heart rate and HRV, respectively. The genes *quo* and *si:dkey-65j6.2* are orthologues of the human *KIAA1755* .

Supplementary Table 8: Effects of CRISPR/Cas9-induced nonsense mutations in both alleles vs. no CRISPR/Cas9-induced mutations on (change in) heart rate variability and heart rate

				Model 1					Model 2				
**Gene**	**Age**	**n**	**Outcome**	**Effect size**	**SE**	**LCI**	**UCI**	**P**	**Effect size**	**SE**	**LCI**	**UCI**	**P**
syt10	2dpf	146	HRV	-0.073	0.205	-0.475	0.329	7.22E-01	-0.128	0.182	-0.485	0.229	4.82E-01
			Heart rate	-0.115	0.199	-0.506	0.276	5.64E-01	-0.103	0.176	-0.449	0.243	5.59E-01
	5dpf	180	HRV	-0.066	0.191	-0.440	0.308	7.30E-01	-0.133	0.157	-0.441	0.174	3.96E-01
			Heart rate	-0.184	0.189	-0.555	0.187	3.31E-01	-0.198	0.152	-0.496	0.101	1.95E-01
	Δ5dpf - 2dpf	123	ΔHRV	0.014	0.198	-0.375	0.403	9.43E-01	-0.082	0.172	-0.420	0.255	6.33E-01
			Δheart rate	-0.228	0.225	-0.668	0.213	3.12E-01	-0.232	0.197	-0.618	0.153	2.37E-01
hcn4	2dpf	183	HRV	0.148	0.399	-0.634	0.930	7.11E-01	0.520	0.344	-0.153	1.194	1.30E-01
			Heart rate	0.695	0.358	-0.006	1.396	5.20E-02	0.674	0.309	0.068	1.280	2.93E-02
	5dpf	212	HRV	-1.349	0.338	-2.010	-0.687	6.48E-05	-0.759	0.294	-1.335	-0.182	9.91E-03
			Heart rate	1.208	0.321	0.579	1.836	1.66E-04	0.444	0.278	-0.100	0.988	1.10E-01
	Δ5dpf - 2dpf	152	ΔHRV	-0.677	0.400	-1.461	0.107	9.04E-02	-0.350	0.344	-1.024	0.324	3.09E-01
			Δheart rate	0.785	0.438	-0.074	1.644	7.34E-02	0.373	0.378	-0.368	1.113	3.24E-01
neo1a	2dpf	70	HRV	0.029	0.343	-0.643	0.702	9.32E-01	0.094	0.317	-0.527	0.714	7.67E-01
			Heart rate	0.102	0.294	-0.473	0.677	7.28E-01	0.216	0.264	-0.302	0.734	4.15E-01
	5dpf	93	HRV	0.089	0.286	-0.473	0.650	7.57E-01	0.140	0.249	-0.348	0.628	5.75E-01
			Heart rate	0.166	0.274	-0.372	0.704	5.45E-01	0.113	0.247	-0.371	0.597	6.47E-01
	Δ5dpf - 2dpf	60	ΔHRV	0.192	0.392	-0.575	0.960	6.23E-01	0.635	0.323	0.002	1.269	4.93E-02
			Δheart rate	0.702	0.342	0.032	1.372	4.02E-02	0.478	0.296	-0.102	1.057	1.07E-01
neo1b	2dpf	80	HRV	0.292	0.308	-0.312	0.896	3.43E-01	0.332	0.290	-0.236	0.901	2.52E-01
			Heart rate	0.153	0.262	-0.360	0.667	5.58E-01	0.225	0.243	-0.251	0.701	3.55E-01
	5dpf	89	HRV	0.053	0.333	-0.600	0.706	8.74E-01	-0.092	0.289	-0.658	0.474	7.50E-01
			Heart rate	0.025	0.325	-0.612	0.661	9.40E-01	-0.073	0.276	-0.614	0.469	7.92E-01
	Δ5dpf - 2dpf	63	ΔHRV	-0.515	0.364	-1.227	0.198	1.57E-01	-0.555	0.308	-1.159	0.049	7.17E-02
			Δheart rate	0.014	0.374	-0.720	0.747	9.71E-01	-0.277	0.331	-0.925	0.372	4.03E-01
quo	2dpf	170	HRV	0.227	0.206	-0.176	0.631	2.70E-01	0.150	0.193	-0.228	0.528	4.36E-01
			Heart rate	-0.104	0.178	-0.454	0.245	5.58E-01	0.006	0.167	-0.321	0.332	9.73E-01
	5dpf	214	HRV	0.240	0.193	-0.139	0.618	2.14E-01	0.203	0.177	-0.144	0.550	2.52E-01
			Heart rate	-0.065	0.192	-0.441	0.310	7.34E-01	0.012	0.163	-0.308	0.332	9.40E-01
	Δ5dpf - 2dpf	146	ΔHRV	0.045	0.250	-0.445	0.535	8.57E-01	0.283	0.214	-0.137	0.703	1.86E-01
			Δheart rate	0.424	0.236	-0.039	0.887	7.27E-02	0.413	0.206	0.009	0.816	4.49E-02
si:dkey-65j6.2	2dpf	60	HRV	0.291	0.298	-0.294	0.876	3.29E-01	0.224	0.281	-0.327	0.775	4.25E-01
			Heart rate	-0.110	0.226	-0.552	0.332	6.27E-01	0.027	0.216	-0.397	0.452	8.99E-01
	5dpf	67	HRV	0.364	0.350	-0.322	1.051	2.98E-01	0.395	0.328	-0.248	1.038	2.29E-01
			Heart rate	0.111	0.298	-0.473	0.695	7.08E-01	0.160	0.275	-0.378	0.698	5.60E-01
	Δ5dpf - 2dpf	50	ΔHRV	-0.648	0.425	-1.480	0.184	1.27E-01	-0.677	0.419	-1.497	0.144	1.06E-01
			Δheart rate	-0.160	0.412	-0.967	0.647	6.97E-01	-0.270	0.415	-1.083	0.543	5.15E-01

(Change in) heart rate variability (HRV) and heart rate were inverse normally transformed before the analysis, so effect sizes and SEs can be interpreted as z-score units. Associations with outcomes are for embryos carrying frameshift and/or premature stop coding introducing mutations in both alleles vs. embryos free from CRISPR/Cas9-induced mutations. Associations were examined using hierarchical linear models (xtmixed in Stata) and were adjusted for time of day and for the weighted number of mutated alleles in the other genes as fixed factors, with larvae nested in batches (random factor). In Model 2, associations were additionally adjusted for (change in) heart rate and HRV, respectively. The genes *quo* and *si:dkey-65j6.2* are orthologues of the human *KIAA1755* .

Supplementary Table 9: Additive effect of CRISPR/Cas9-induced mutations on body size

			2dpf						5dpf					
**Outcome**	**Factor**	**Gene**	**n**	**Effect size**	**SE**	**LCI**	**UCI**	**P**	**n**	**Effect size**	**SE**	**LCI**	**UCI**	**P**
		gngt1		-0.120	0.115	-0.346	0.106	2.97E-01		0.043	0.115	-0.182	0.268	7.05E-01
		syt10		0.147	0.080	-0.009	0.303	6.54E-02		0.095	0.076	-0.053	0.243	2.09E-01
		rgs6		-0.444	0.179	-0.795	-0.094	1.31E-02		-0.467	0.181	-0.822	-0.113	9.65E-03
		hcn4		-0.131	0.101	-0.330	0.067	1.96E-01		0.080	0.094	-0.104	0.265	3.95E-01
		hcn4l		0.126	0.200	-0.265	0.518	5.28E-01		-0.075	0.192	-0.452	0.301	6.95E-01
	fixed	*neo1a*		-0.029	0.110	-0.244	0.185	7.89E-01		-0.136	0.108	-0.347	0.076	2.09E-01
Length		*neo1b*	332	0.075	0.122	-0.163	0.314	5.37E-01	242	0.045	0.127	-0.205	0.294	7.26E-01
		quo		-0.008	0.083	-0.171	0.156	9.27E-01		-0.026	0.082	-0.186	0.134	7.52E-01
		si:dkey-65j6.2		0.045	0.107	-0.165	0.254	6.77E-01		-0.087	0.105	-0.293	0.119	4.08E-01
		time of day		0.111	0.042	0.029	0.193	7.88E-03		0.091	0.047	-0.001	0.183	5.25E-02
		intercept		-0.490	0.371	-1.216	0.237	1.86E-01		-0.131	0.449	-1.011	0.750	7.71E-01
	random	variation by batch		0.395	0.129	0.208	0.748	-		0.689	0.227	0.361	1.315	-
		residual		0.897	0.035	0.830	0.968	-		0.703	0.032	0.643	0.770	-
		gngt1		0.025	0.110	-0.190	0.240	8.20E-01		-0.091	0.137	-0.360	0.178	5.08E-01
		syt10		0.052	0.076	-0.096	0.201	4.89E-01		0.048	0.090	-0.129	0.225	5.99E-01
		rgs6		-0.031	0.168	-0.361	0.299	8.55E-01		-0.462	0.223	-0.899	-0.024	3.86E-02
		hcn4		-0.022	0.096	-0.210	0.167	8.22E-01		-0.090	0.110	-0.305	0.126	4.14E-01
		hcn4l		0.066	0.188	-0.303	0.435	7.25E-01		0.097	0.222	-0.338	0.531	6.63E-01
	fixed	*neo1a*		0.208	0.105	0.002	0.414	4.76E-02		-0.032	0.125	-0.277	0.214	8.01E-01
Dorsal area		*neo1b*	322	0.003	0.117	-0.226	0.232	9.81E-01	234	-0.139	0.154	-0.441	0.163	3.66E-01
		quo		0.052	0.080	-0.105	0.210	5.16E-01		0.267	0.095	0.081	0.452	4.92E-03
		si:dkey-65j6.2		-0.184	0.103	-0.386	0.019	7.50E-02		-0.274	0.123	-0.515	-0.034	2.54E-02
		time of day		-0.052	0.040	-0.131	0.026	1.89E-01		-0.072	0.053	-0.176	0.032	1.74E-01
		intercept		-0.049	0.365	-0.765	0.668	8.94E-01		0.428	0.406	-0.369	1.225	2.92E-01
	random	variation by batch		0.437	0.138	0.236	0.810	-		0.300	0.129	0.130	0.696	-
		residual		0.842	0.033	0.779	0.910	-		0.824	0.039	0.751	0.903	-
		gngt1		0.052	0.124	-0.192	0.295	6.76E-01		-0.172	0.119	-0.405	0.060	1.47E-01
		syt10		0.086	0.087	-0.084	0.255	3.24E-01		-0.007	0.082	-0.167	0.153	9.30E-01
		rgs6		-0.063	0.182	-0.419	0.293	7.27E-01		-0.074	0.175	-0.417	0.269	6.72E-01
		hcn4		-0.137	0.106	-0.344	0.070	1.95E-01		-0.115	0.102	-0.314	0.084	2.57E-01
		hcn4l		0.053	0.208	-0.354	0.460	7.99E-01		0.195	0.200	-0.196	0.586	3.29E-01
	fixed	*neo1a*		0.235	0.112	0.015	0.454	3.61E-02		-0.034	0.122	-0.272	0.204	7.80E-01
Lateral area		*neo1b*	300	-0.051	0.127	-0.300	0.199	6.89E-01	258	-0.138	0.148	-0.428	0.151	3.48E-01
		quo		0.139	0.088	-0.033	0.312	1.13E-01		0.262	0.092	0.080	0.443	4.66E-03
		si:dkey-65j6.2		-0.248	0.111	-0.466	-0.030	2.57E-02		-0.402	0.117	-0.632	-0.173	5.79E-04
		time of day		-0.047	0.044	-0.134	0.040	2.91E-01		-0.074	0.050	-0.172	0.023	1.35E-01
		intercept		-0.001	0.355	-0.697	0.695	9.97E-01		0.595	0.415	-0.217	1.408	1.51E-01
	random	variation by batch		0.265	0.104	0.123	0.570	-		0.440	0.154	0.221	0.876	-
		residual		0.908	0.038	0.837	0.985	-		0.796	0.035	0.729	0.868	-
		gngt1		0.088	0.110	-0.129	0.304	4.27E-01		-0.187	0.129	-0.441	0.066	1.47E-01
		syt10		0.052	0.076	-0.097	0.202	4.93E-01		-0.023	0.083	-0.186	0.141	7.87E-01
		rgs6		0.093	0.174	-0.249	0.435	5.93E-01		-0.042	0.180	-0.394	0.310	8.13E-01
		hcn4		-0.014	0.096	-0.203	0.174	8.80E-01		0.017	0.102	-0.182	0.217	8.64E-01
		hcn4l		-0.033	0.185	-0.395	0.330	8.59E-01		0.092	0.229	-0.357	0.541	6.87E-01
	fixed	*neo1a*		0.314	0.103	0.113	0.515	2.23E-03		0.033	0.117	-0.196	0.262	7.76E-01
Volume		*neo1b*	299	-0.062	0.111	-0.279	0.154	5.73E-01	199	0.105	0.143	-0.176	0.386	4.64E-01
		quo		0.153	0.078	0.000	0.305	4.98E-02		0.150	0.091	-0.028	0.328	9.82E-02
		si:dkey-65j6.2		-0.239	0.100	-0.435	-0.042	1.74E-02		-0.398	0.116	-0.626	-0.170	6.23E-04
		time of day		-0.065	0.040	-0.144	0.015	1.10E-01		-0.075	0.052	-0.177	0.027	1.50E-01
		intercept		-0.153	0.355	-0.849	0.544	6.67E-01		0.198	0.448	-0.679	1.075	6.58E-01
	random	variation by batch		0.427	0.136	0.229	0.796	-		0.572	0.196	0.292	1.120	-
		residual		0.806	0.033	0.743	0.874	-		0.718	0.037	0.650	0.794	-

Dorsal body surface area, lateral body surface area, and body volume were normalized for body length before the analysis, and outcomes were inverse normally transformed, so effect sizes and SEs can be interpreted as z-score units. Associations of outcomes with the weighted number of mutated alleles in the main transcript of each targeted zebrafish orthologue were examined using hierarchical linear models (xtmixed in Stata). Associations were adjusted for time of day and for the weighted number of mutated alleles in the other genes as fixed factors, with embryos nested in batches (random factor). The genes *quo* and *si:dkey-65j6.2* are orthologues of the human *KIAA1755* .

Supplementary Table 10: Effect of CRISPR/Cas9-induced nonsense mutations in both alleles vs. no CRISPR/Cas9-induced mutations on body size

		2dpf						5dpf					
**Gene**	**Outcome**	**n**	**Effect size**	**SE**	**LCI**	**UCI**	**P**	**n**	**Effect size**	**SE**	**LCI**	**UCI**	**P**
syt10	Length	208	0.207	0.174	-0.135	0.548	2.35E-01	153	0.174	0.182	-0.182	0.530	3.38E-01
	Dorsal area	200	0.022	0.174	-0.320	0.363	9.01E-01	145	0.101	0.204	-0.299	0.501	6.20E-01
	Lateral area	182	0.002	0.200	-0.390	0.393	9.93E-01	155	-0.122	0.193	-0.501	0.257	5.28E-01
	Volume	186	-0.028	0.173	-0.366	0.311	8.73E-01	128	-0.170	0.201	-0.564	0.224	3.98E-01
hcn4	Length	241	0.026	0.306	-0.573	0.625	9.33E-01	177	0.130	0.291	-0.439	0.700	6.54E-01
	Dorsal area	233	-0.020	0.309	-0.626	0.586	9.49E-01	171	0.440	0.319	-0.185	1.065	1.67E-01
	Lateral area	217	0.154	0.321	-0.475	0.783	6.31E-01	183	0.300	0.326	-0.339	0.940	3.57E-01
	Volume	216	0.264	0.303	-0.330	0.858	3.83E-01	141	0.474	0.280	-0.075	1.024	9.06E-02
neo1a	Length	107	-0.188	0.263	-0.703	0.328	4.75E-01	77	-0.317	0.317	-0.939	0.305	3.17E-01
	Dorsal area	104	0.707	0.246	0.224	1.189	4.08E-03	75	0.242	0.340	-0.424	0.907	4.76E-01
	Lateral area	97	-0.072	0.280	-0.621	0.476	7.96E-01	84	0.202	0.356	-0.496	0.901	5.70E-01
	Volume	94	0.516	0.266	-0.005	1.037	5.23E-02	69	-0.164	0.261	-0.676	0.347	5.29E-01
neo1b	Length	117	0.241	0.311	-0.369	0.851	4.38E-01	76	0.378	0.339	-0.286	1.042	2.64E-01
	Dorsal area	112	-0.056	0.296	-0.635	0.523	8.49E-01	72	-0.206	0.430	-1.050	0.637	6.32E-01
	Lateral area	106	0.343	0.336	-0.315	1.001	3.07E-01	74	0.209	0.394	-0.564	0.982	5.96E-01
	Volume	110	0.170	0.284	-0.386	0.726	5.49E-01	62	0.181	0.373	-0.550	0.913	6.27E-01
quo	Length	241	-0.115	0.182	-0.471	0.241	5.27E-01	180	0.078	0.171	-0.256	0.413	6.46E-01
	Dorsal area	235	0.238	0.178	-0.110	0.587	1.80E-01	175	0.545	0.210	0.133	0.957	9.52E-03
	Lateral area	223	0.244	0.198	-0.144	0.632	2.18E-01	188	0.394	0.212	-0.021	0.809	6.30E-02
	Volume	218	0.331	0.174	-0.010	0.671	5.70E-02	146	0.175	0.197	-0.211	0.560	3.75E-01
si:dkey-65j6.2	Length	80	0.221	0.325	-0.416	0.858	4.96E-01	53	-0.107	0.345	-0.782	0.569	7.57E-01
	Dorsal area	78	-0.110	0.266	-0.631	0.412	6.80E-01	51	-0.566	0.317	-1.188	0.056	7.45E-02
	Lateral area	74	-0.489	0.295	-1.067	0.089	9.72E-02	57	-0.622	0.389	-1.384	0.139	1.09E-01
	Volume	75	-0.300	0.262	-0.813	0.212	2.51E-01	44	-0.732	0.338	-1.395	-0.070	3.03E-02

Dorsal body surface area, lateral body surface area, and body volume were normalized for body length before the analysis, and all outcomes were inverse normally transformed, so effect sizes and SEs can be interpreted as z-score units. Associations with outcomes are for embryos carrying CRISPR/Cas9-induced nonsense mutations in both alleles vs. embryos free from CRISPR/Cas9-induced mutations. Associations were examined using hierarchical linear models (xtmixed in Stata) and were adjusted for time of day and for the weighted number of mutated alleles in the other genes as fixed factors, with embryos nested in batches (random factor). The genes *quo* and *si:dkey-65j6.2* are orthologues of the human *KIAA1755*.

Supplementary Table 11: Transcripts with at least 75% sequence similarity to the zebrafish *hcn4* cDNA sequence

Gene	ENSDARG stable ID	BLAST Score	%ID	Human orthologue	ENSG stable ID	Target %id	Query %id
hcn4l	ENSDARG00000074419	479	85.20	*HCN4*	ENSG00000138622	59.96	50.04
CABZ01086574.1	ENSDARG00000116404	277	80.09	*HCN2*	ENSG00000099822	60.30	22.72
hcn2b	ENSDARG00000061665	249	85.63	*HCN2*	ENSG00000099822	-	-
hcn3	ENSDARG00000027192	200	82.35	*HCN3*	ENSG00000143630	-	-
hcn1	ENSDARG00000104480	160	78.42	*HCN1*	ENSG00000164588	72.25	73.71

Zebrafish genes with transcripts showing at least 75% sequence similarity to the main zebrafish *hcn4* transcript were selected for a qRT-PCR experiment to explore possible compensatory responses to CRISPR/Cas9 mutagenesis of *hcn4* . BLAST score represents the highest score for a transcript of that zebrafish gene against the *hcn4* cDNA sequence. 'Human orthologue' shows for which human gene that zebrafish gene was flagged as an orthologue in Ensembl. Target %id is the percentage of the orthologous sequence that matches the human sequence (Ensembl); Query %id is the percentage of the human sequence matching the sequence of the orthologue.

Supplementary Table 12: qRT-PCR target sites, primers and experimental conditions

Gene	Ensembl gene ID	Site	Forward Primer Sequence (5’- 3’)	Reverse Primer Sequence (5’- 3’)	Product size (bp)	Primer efficiency	R^2	slope	Melting Temperature (°C)
hcn4	ENSDARG00000061685	CRISPR/Cas9 cut site	CTCCTGGAGCCGCGGGTG	TGCTCGATGCCTTCCAGCTT	139	111.62	0.983	-3.072	92.5
		last exon	GGCCGCTTTTATGAGGATTT	GGCCTGGAGAAGTCTGTACG	178	118.58	0.991	-2.495	95.0
hcn4l	ENSDARG00000074419	CRISPR/Cas9 cut site	TCTTCTAATGGTGGGCAACC	CTGCGCAAGTACCTGACCTT	210	116.38	0.960	-2.983	84.0
		last exon	GGCTTGGCTCACTGAAAGAC	GCGGTGTGGAGGTAGATGAT	171	117.26	0.966	-2.968	95.0
CABZ01086574.1	ENSDART00000192280		GAGTCAGCGGAGGTGTATCG	TGCGCTTGCTAGGTCATAGG	156	105.86	0.962	-3.189	82.0
hcn2b	ENSDARG00000061665		AAACCACCACCCCTTGGATT	CACCGGGATGGATGAGACAAA	188	154.33	0.998	-2.467	81.0
hcn3	ENSDARG00000027192		TGTGTTGGTCATCCACCCCT	CGGTGACGGATGCTTTTCAA	255	132.93	0.994	-2.723	84.5
hcn1	ENSDARG00000104480		AAGACTTCCCTCCCGATTGC	TCCAGAGGTCGGACATGCTA	153	159.97	0.989	-2.410	85.0
mob4	ENSDARG00000056085		CACCCGTTTCGTGATGAAGTACAA	GTTAAGCAGGATTTACAATGGAG	297	115.52	0.979	-2.999	85.0

Supplementary Table 13: Effects of targeting *hcn4*, *hcn4l*, or *hcn4* and *hcn4l* using CRISPR/Cas9 on the expression of genes with >75% sequence similarity to the main zebrafish *hcn4* transcript

			Model 1						Model 2				
**Outcome**	**Exposure**	**n**	**Effect size**	**SE**	**LCI**	**UCI**	**P**	**n**	**Effect size**	**SE**	**LCI**	**UCI**	**P**
hcn4 - on target	*hcn4*	68	-0.226	0.049	-0.325	-0.127	2.40E-05						
	hcn4l		0.054	0.048	-0.041	0.150	2.62E-01						
	hcn4 & *hcn4l*		-0.230	0.040	-0.310	-0.150	2.90E-07						
hcn4 - last exon	*hcn4*	69	0.028	0.072	-0.115	-0.172	6.96E-01						
	hcn4l		0.074	0.069	-0.064	0.212	2.88E-01						
	hcn4 & *hcn4l*		-0.013	0.578	-0.128	0.103	8.27E-01						
hcn4l - on target	*hcn4*	68	0.041	0.062	-0.082	0.165	5.06E-01						
	hcn4l		-0.048	0.611	-0.170	0.074	4.36E-01						
	hcn4 & *hcn4l*		-0.025	0.050	-0.124	0.075	6.20E-01						
hcn4l - last exon	*hcn4*	69	0.064	0.084	-0.103	0.232	4.47E-01						
	hcn4l		-0.019	0.081	-0.180	0.142	8.19E-01						
	hcn4 & *hcn4l*		0.047	0.067	-0.088	0.182	4.91E-01						
CABZ01086574.1	*hcn4*	64	0.444	0.193	0.058	0.830	2.50E-02	63	0.056	0.109	-0.162	0.274	6.10E-01
	hcn4l		0.045	0.203	-0.362	0.452	8.24E-01		0.016	0.110	-0.204	0.236	8.83E-01
	hcn4 & *hcn4l*		0.148	0.149	-0.150	0.447	3.24E-01		0.117	0.008	-0.044	0.279	1.50E-01
hcn2b	*hcn4*	68	0.177	0.085	0.007	0.347	4.20E-02						
	hcn4l		-0.006	0.082	-0.169	0.158	9.43E-01						
	hcn4 & *hcn4l*		0.041	0.070	-0.099	0.180	5.62E-01						
hcn3	*hcn4*	66	0.034	0.068	-0.102	0.171	6.17E-01	65	0.035	0.041	-0.046	0.117	3.86E-01
	hcn4l		0.074	0.066	-0.057	0.206	2.64E-01		-0.022	0.040	-0.102	0.059	5.93E-01
	hcn4 & *hcn4l*		-0.038	0.056	-0.151	0.075	5.05E-01		-0.035	0.034	-0.102	0.032	3.04E-01
hcn1	*hcn4*	68	0.376	0.225	-0.073	0.825	9.90E-02	67	0.001	0.166	-0.330	0.333	9.93E-01
	hcn4l		0.073	0.216	-0.360	0.505	7.39E-01		0.024	0.153	-0.282	0.331	8.74E-01
	hcn4 & *hcn4l*		0.163	0.182	-0.200	0.526	3.72E-01		0.126	0.129	-0.131	0.383	3.32E-01

All samples consisted of pooled tissue from five 5-day-old embryos. Three technical replicates per sample were used to calculate an average quantification cycle (Cq) for each sample, which were used to calculate a gene expression ratio (GER) for each sample using non-injected controls as a calibrator, and a reference gene (*mob4*) for normalization, using the Pfaffl method. GERs were used as outcomes in multiple linear regression analyses, to examine the effect of targeting *hcn4* (n=10), *hcn4l* (n=12), or *hcn4* & *hcn4l* (n=21) using CRISPR/Cas9 as compared with control samples that had either been micro-injected with Cas9 mRNA only, or *hcn4* & *hcn4l* gRNA only at the single-cell stage (n=26). Since the experiment was performed twice to reach an adequate sample size, associations were adjusted for batch. Model 1 makes use of all samples; while in Model 2, observations with a GER outside the mean ± 5·SD interval were excluded from the analysis. The latter resulted in the exclusion of one sample of *hcn4*-targeted embryos from the analysis for *CABZ01086574.1* (likely an orthologue of *HCN2*) and *hcn1*; and one sample of *hcn4l*-targeted embryos from the analysis for *hcn3*.

Supplementary Table 14: Druggability of interacting partners of putative causal genes

Candidate	Interaction partner	DGIdb categories	Drug or compound name	Category
SYT10	*BOP1*	Tumor supressor		
	SNAP23	Druggable genome		
	SNAP25	Druggable genome	Diazoxide	Anti-hypertensive agents
			Botulinum toxin type A	Neuromuscular blocking agents
	SNAP29	Druggable genome		
	SNAP47			
	SYT6			
RGS6	*CCT6B*	Transporter		
	DMAP1	DNA repair, histone modification, transcription factor binding		
	GNAI1	Tumor suppressor	CHEMBL384759	experimental
	GNAI2	Serine-threonine kinase		
	GNAI3			
	GNAO1	Drug resistance		
	GNAT3			
	GNB3		Lovastatin, Simvastatin, Cerivastatin	Anti-cholesterimic agents
			Olanzapine	Anti-psychotic agents
			Hydrocholorothiazide	Anti-hypertensive agents
	GNB5	Transporter, Ion channel		
	PDCL			
	RGS11			
	RGS7			
	RGS9			
	STMN2			
HCN4	*HCN1*	Druggable genome, Transporter, Ion channel	Ivabradine, Cilobradine, Zatebradine, CHEMBL2052019	Channel blocker
	HCN2	Druggable genome, Transporter, Ion channel	Cilobradine, Zatebradine, CHEMBL2052019	Channel blocker
	HCN3	Druggable genome, Transporter, Ion channel	Ivabradine, Cilobradine, Zatebradine, CHEMBL2052019	Channel blocker
	PEX5L			
KIAA1755	-			

Interaction partners of human candidate genes were identified using STRING and Genemania, and their classification was identified using the Drug gene interaction database (DGIdb). A selection of compounds and FDA-approved drugs was distilled from DGIdb.

Supplementary Table 15: Effect of 24h of ivabradine treatment on heart rate variability and heart rate in 5dpf zebrafish embryos

			Model 1						Model 2					
Outcome	Factor	Exposure	n	Effect size	SE	LCI	UCI	P	n	Effect size	SE	LCI	UCI	P
HRV	fixed	ivabradine (10 µM)	109	0.328	0.179	-0.022	0.678	6.65E-02	109	0.051	0.165	-0.273	0.374	7.58E-01
		ivabradine (25 µM)		0.905	0.172	0.568	1.242	1.45E-07		0.258	0.190	-0.115	0.631	1.76E-01
		heart rate (in SD)		-	-	-	-	-		-0.523	0.093	-0.706	-0.340	2.02E-08
		time of day		-0.057	0.071	-0.197	0.083	4.24E-01		0.105	0.069	-0.031	0.240	1.31E-01
		intercept		-0.541	0.283	-1.096	0.015	5.64E-02		-0.783	0.253	-1.279	-0.286	2.00E-03
	random	variation by batch		-	0.000	0.000	0.001	-		-	0.000	0.000	1.009	-
		residual		-	0.051	0.660	0.860	-		-	0.045	0.581	0.758	-
Heart rate	fixed	ivabradine (10 µM)	118	-0.524	0.158	-0.835	-0.214	9.35E-04	109	-0.401	0.142	-0.678	-0.123	4.68E-03
		ivabradine (25 µM)		-1.373	0.154	-1.675	-1.071	5.29E-19		-0.872	0.151	-1.169	-0.576	8.07E-09
		HRV (in SD)		-	-	-	-	-		-0.415	0.075	-0.563	-0.267	3.63E-08
		time of day		0.251	0.072	0.110	0.392	4.69E-04		0.274	0.061	0.154	0.395	8.28E-06
		intercept		-0.251	0.301	-0.841	0.338	4.04E-01		-0.655	0.246	-1.137	-0.173	7.76E-03
	random	variation by batch		-	0.113	0.114	0.611	-		-	0.089	0.038	0.488	-
		residual		-	0.046	0.611	0.794	-		-	0.041	0.512	0.672	-

Heart rate variability (HRV) and heart rate were inverse normally transformed before the analysis, so effect sizes and SEs can be interpreted as z-score units. Associations of outcomes with exposure to 10 or 25 µM ivabradine in DMSO vs. DMSO only were examined using hierarchical linear models (xtmixed in Stata). Associations were adjusted for time of day as fixed factors, with embryos nested in batches (random factor). In Model 2, associations were additionally adjusted for heart rate and HRV, respectively.
